# Luminescent Iridium–Terpyridine Complexes with Various Bis-Cyclometalated Ligands

**DOI:** 10.3390/molecules30010193

**Published:** 2025-01-06

**Authors:** Ko Ikeda, Natsumi Yano, Makoto Handa, Yusuke Kataoka

**Affiliations:** Department of Chemistry, Graduate School of Natural Science and Technology, Shimane University, 1060, Nishikawatsu, Matsue 690-8504, Shimane, Japan

**Keywords:** cyclometalated iridium complexes, luminescent properties, crystal structures, photophysical properties, terpyridine ligands, supramolecular interactions

## Abstract

A series of luminescent bis-cyclometalated iridium complexes with 2,2′:6′,2″-terpyridine (tpy), [Ir(*C^N*)_2_(tpy)]PF_6_ (*C^N* = 2-phenylpyridinate (ppy) for **1**; benzo[h]quinolinate (bzq) for **2**; 1-phenylisoquinolinate (piq) for **3**; and 2-phenylbenzothiazolate (pbt) for **4**), have been synthesized and structurally characterized. Single-crystal X-ray diffraction analyses reveal that the tpy ligands of **1**–**4** are coordinated to the iridium center in a bidentate fashion, and the uncoordinated pendant pyridine rings in the tpy ligands of **1**–**4** form intramolecular π-π stacking interactions with a phenyl moiety of *C^N* ligands. In addition, the pendant pyridine ring in the tpy ligand of **1** forms an intramolecular hydrogen bonding interaction, unlike in **2**–**4**. Of interest, the photophysical properties of **1**–**4** are strongly influenced by the *C^N* ligands; **1** shows a luminescence band at 572 nm, with a short lifetime (τ) value of 80 nsec and a lower absolute luminescence quantum yield (Φ) of 3.72%, whereas **3** exhibits an intense luminescence band at 588 nm with a long τ value of 1965 nsec and a moderate Φ value of 9.57%. The density functional theory calculations revealed that the luminescence originates from the triplet metal–ligand to ligand charge transfer (^3^MLL′CT) excited state.

## 1. Introduction

Bis-cyclometalated iridium(III) complexes, formulated as [Ir(*C^N*)_2_(*N^N*)]^+^, in which *C^N* and *N^N* are bidentate cyclometalated ligands, such as 2-phenylpyridinate (ppy), and diimine ligands, such as 2,2′-bipyridine (bpy), respectively, are well-known series of highly luminescent organometallic compounds [1,2] and have been widely investigated not only for their intrinsic photophysical properties [3,4,5] but also for their remarkable photochemical applications, such as emissive dopants for organic light-emitting diodes (OLEDs) [6,7,8,9]; photosensitizers for artificial photosynthesis [10,11,12,13]; photo-redox reactions [14,15,16]; and probes for ions [17] and cell imaging [18,19]. The excellent photophysical properties of these iridium complexes are derived from both the strong spin–orbit coupling effect of the iridium center and the strong ligand-field nature due to the cyclometalating Ir-C bonds [20]. In addition, the luminescence generally originates from triplet metal–ligand to ligand charge transfer (^3^MLL′CT) excited states, i.e., spin-forbidden (phosphorescence) transitions from the *N^N* moiety to the Ir(*C^N*)_2_ moiety [7,14,21]. In general, the highest occupied molecular orbital (HOMO) and lowest unoccupied molecular orbital (LUMO) of these iridium complexes are localized on the [Ir(*C^N*)_2_] and *N^N* moieties, respectively; hence, the photophysical properties of these iridium complexes can be finely tuned by controlling the electronic structures (i.e., orbital energies) of both or either *C^N* and *N^N* ligands through structural modifications [22,23,24].

**Scheme 1:**
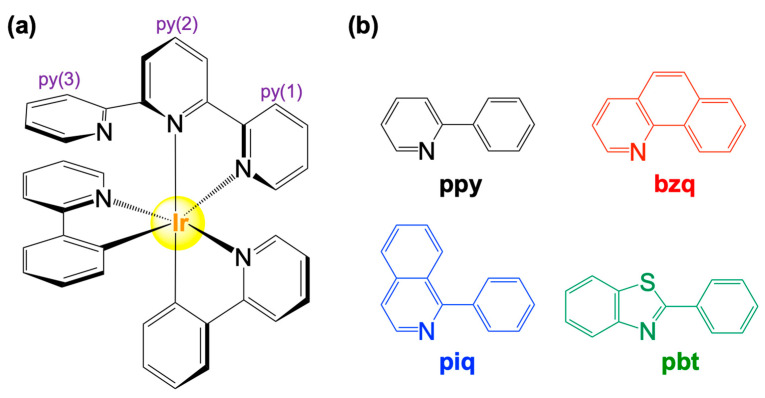
Molecular structures: (**a**) [Ir(ppy)_2_(tpy)]^+^, with the pyridyl rings numbered as py(1), py(2), and py(3); (**b**) cyclometalated ligands used in this study.

In the [Ir(*C^N*)_2_(*N^N*)]^+^ complexes, the *N^N* ligands with an aryl group at the 6-position of the bpy derivatives have been reported to form unique intramolecular, supramolecular interactions between the aryl group and the cyclometalated ligands (see Scheme 1a) [25,26,27,28,29,30,31,32]. For example, a bis-cyclometalated iridium complex with a 2,2′:6′,2″-terpyridine (tpy) ligand [Ir(ppy)_2_(tpy)]PF_6_ (**1**), which forms an intramolecular face-to-face π-π stacking interaction and a CH(ppy)-N(tpy) hydrogen bonding interaction between the non-coordinated pyridine of the tpy ligand and the cyclometalated phenyl ring, exhibits an orange luminescence at λ_max_ = 590 nm, with a lifetime (τ) of 68 ns and a quantum yield (Φ) of 1.7% in degassed CH_2_Cl_2_ solution [29]. Interestingly, **1** has been shown to function as an emitter in light-emitting electrochemical devices, with relatively low efficiency but rapid turn-on times. Preparations and characterizations of bis-cyclometalated iridium complexes with several 4-substituted tpy (R-tpy) ligands have already been performed, revealing that their iridium complexes also form both intramolecular π-π stacking and CH(ppy)-N(tpy) hydrogen bonding interactions between the non-coordinated pyridine of the tpy ligand and the cyclometalated phenyl ring, and the luminescent properties of these iridium complexes change significantly depending on the substituents at the 4-position of tpy ligands [30,31,32]. On the other hand, the influences of the *C^N* cyclometalated ligands in [Ir(*C^N*)_2_(tpy)]^+^ on their molecular structures (including intramolecular interactions), electronic structures, and electrochemical and photophysical properties have not been thoroughly investigated. 

In this study, we performed experimental and theoretical investigations into [Ir(*C^N*)_2_(tpy)]PF_6_ complexes bearing four different *C^N* ligands (*C^N* = 2-phenylpyridinate (ppy) for **1**; benzo[h]quinolinate (bzq) for **2**; 1-phenylisoquinolinate (piq) for **3**; and 2-phenylbenzothiazolate (pbt) for **4**) to clarify the influences of *C^N* cyclometalated ligands in [Ir(*C^N*)_2_(tpy)]PF_6_ complexes on their molecular geometries, electronic structures, and photophysical and electrochemical properties. The crystal structures of **1**–**4** were determined, and it was clarified that the structural distortions of the tpy ligands in their iridium complexes change depending on the type of *C^N* ligands. Furthermore, it was found that the photophysical properties of the iridium complexes are also strongly affected by the type of *C^N* ligands.

## 2. Results

### 2.1. Synthesis and Characterizations

Complexes **1**–**4** were prepared by reflux reactions of chloride-bridged cyclometalated iridium dimers, [Ir(*C^N*)_2_Cl]_2_, with 2.2 equiv. of tpy in a 1,2-dichloroethane/methanol (1:1) solution, followed by washing with diethyl ether, the addition of excess ammonium hexafluorophosphate (NH_4_PF_6_) in aqueous solution, and drying under vacuum at 393 K. Yellow or yellowish orange powders of **1**–**4** were obtained in the yields of 80.6, 89.7, 81.7, and 91.5%, respectively, which are much higher than that for the previously reported **1** (47.6%), prepared by microwave synthesis and purified using column chromatography [29]. Complexes **1**–**4** were insoluble in water but highly soluble in chloroform (CHCl_3_) and propylene carbonate (PC). Hence, the latter organic solvents were used for the photophysical and electrochemical analyses, respectively.

The positive electrospray ionization time-of-flight mass spectroscopy (ESI-TOF-MS) of **1**–**4** showed peaks at 734.1890, 782.1890, 834.2198, and 846.1347 *m*/*z*, respectively, corresponding to the [M]^+^ values of **1**–**4** (734.1892, 782.1893, 834.2206, and 846.1331 *m*/*z*, respectively). As shown in Figures S1–S4 in the Supplementary Materials, the shapes of the isotope distributions of **1**–**4** are consistent with the simulated ones. ^1^H NMR spectra of **1**–**4** in DMSO-*d*_6_ were complicated because the molecular structures of the complexes did not have asymmetric centers. Considering that 27, 27, 31, and 27 signal peaks attributed to the *C^N* and tpy ligands were observed for **1**–**4**, respectively, the target compounds of **1**–**4** were considered to have been obtained (see Figures S5–S8). Attenuated total reflection-Fourier transform infrared (ATR-FT-IR) spectroscopy of **1**–**4** showed the strong P-F vibrations of the PF_6_^−^ anion at 840–843 cm^−1^. In the elemental analyses, the observed CHN values of **1**–**4** were consistent with the calculated values.

### 2.2. Crystal Structures of ***1***–***4***

**Figure 1:**
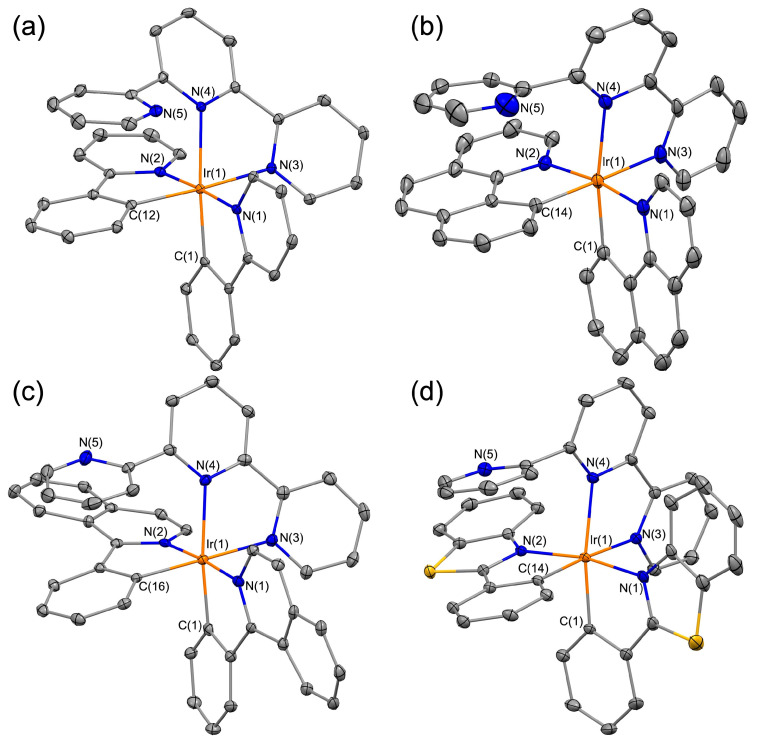
Crystal structures of (**a**) **1**, (**b**) **2**, (**c**) **3**, and (**d**) **4** [Ir: orange, C: glay, N: blue, and S: yellow]. Thermal ellipsoids were set at a 30% probability level, and hydrogen atoms, PF_6_^−^ anions, and solvent molecules were omitted for clarity.

Single crystals suitable for the X-ray diffraction analyses of **1**–**4** were grown by the slow diffusion of hexane into an acetone solution of **1**, hexane into an EtOH solution of **2**, diethyl ether into a MeOH solution of **3**, and toluene into a CH_2_Cl_2_ solution of **4**. The diffraction analyses of **1** and **3** were performed at 100 K using a laboratory SCXRD diffractometer, whereas those of **2** and **4** were performed at 100 K using the BL02B1 beamline at the SPring-8 synchrotron facility in Japan. Further, **1** and **4** crystallized in the monoclinic system with space group *P* 1 2_1_/*c* 1, whereas **2** and **3** crystallized in the orthorhombic system with space group *Pnma* and the monoclinic system with space group *P* 1 2_1_/*n* 1, respectively. Figure 1 shows the crystal structures of **1**–**4**. The bond lengths and angles of the primary coordination sphere of the iridium centers in **1**–**4** are summarized in Tables S1–S4. To simplify the descriptions of the molecular structures of **1**–**4**, the three pyridyl rings of the tpy ligand are referred to as py(1)-py(3). For example, as shown in Scheme 1, the pendant pyridyl ring, whose nitrogen atom is uncoordinated to the iridium center, is referred to as py(3).

The asymmetric units of **1**, **3**, and **4** consist of one [Ir(*C^N*)_2_(tpy)]^+^ cation and one PF_6_^−^ anion, whereas that of **2** consists of one [Ir(bzq)_2_(tpy)]^+^ cation and two half PF_6_^−^ anions, which are located in special positions. The [Ir(*C^N*)_2_(tpy)]^+^ cations in **1**–**4** have two cyclometalating ligands and one tpy ligand in a pseudo-octahedral coordination geometry around the iridium center. In the Ir(*C^N*)_2_ fragment moieties, the nitrogen atoms and cyclometalated carbon atoms in two *C^N* ligands are located in the *trans* and *cis* positions, respectively, with respect to the iridium center, and the Ir-C_(*C^N*)_ and Ir-N_(*C^N*)_ bond lengths of **1**–**4** are in the range of 1.999–2.022 and 2.035–2.065 Å, respectively, which are close to those of previously reported [Ir(ppy)_2_(R-tpy)]^+^ complexes (Ir-C_(*ppy*)_ = 2.00–2.03 Å, Ir-N_(*ppy*)_ = 2.04–2.05 Å) [30] and [Ir(ppy)_2_(bpy)]PF_6_ (Ir-C_(*ppy*)_ = 2.014 Å, Ir-N_(*ppy*)_ = 2.045 Å) [33]. The py(1) and py(2) rings in the tpy ligands of **1**–**4** are coordinated to an iridium center with a distorted ring structure; the Ir(1)-N(4)_[py(2)]_ bond lengths (2.199–2.246 Å) in **1**–**4** are sizably longer than the Ir(1)-N(3)_[py(1)]_ bond lengths (2.126–2.141 Å). Interestingly, the supramolecular interactions between the uncoordinated py(3) ring in tpy and the *C^N* ligands of **1**–**4** were strongly influenced by the type of *C^N* ligands. In **1**, the py(3) ring forms (a) face-to-face π-π stacking interactions with a ppy ligand (centroid distance = 3.486 Å) and (b) a hydrogen bonding interaction with another ppy ligand (CH_(*ppy*)_-N(5)_[py(3)]_ = 2.571 Å), as reported previously. On the other hand, the py(3) ring in **2**, which has a similar structural orientation as **1**, forms a π-π stacking interaction with one bzq ligand but does not form a hydrogen bond with another bzq ligand; the CH_(bzq)_-N(5)_[py(3)]_ distance is 4.176 Å. The py(3) rings of **3** and **4,** which take the opposite orientation relative to those of **1** and **2**, i.e., the N(5) atom faces outward from the molecule, form the π-π stacking interaction with one piq or pbt ligand, respectively, but do not form the hydrogen bond with another piq or pbt ligand. The pyridine-pyridine rotation angles for the tpy values of **1**–**4** are summarized in Table 1. The most distorted tpy feature, which is the largest py(1)-py(2) rotation angle, was found in **1**, resulting in a small py(2)-py(3) rotation angle. The smallest py(1)-py(2) rotation angle, that is, a nearly the coplanar Ir(1)-py(1)-py(2) arrangement, was observed for **4**, resulting in a large py(2)-py(3) rotation angle.

### 2.3. Optimized Geometries and Electronic Structures

To investigate the molecular and electronic structures of **1**–**4** in detail, density functional theory (DFT) calculations with the dispersion collected hybrid B3LYP-D3BJ functional were performed for **1**–**4** at the ground singlet (S_0_) and the lowest excited triplet (T_1_) states in CHCl_3_ (using the polarizable continuum model). The obtained optimized geometries of **1**–**4** are shown in Figures S9–S12, and the bond lengths and angles of the primary coordination spheres of the iridium centers in their structures are summarized in Tables S1–S4.

In the S_0_ state, the primary coordination spheres of the iridium centers in the optimized geometries of **1**–**4** agreed well with their crystal structures, with differences of less than 0.04 Å. The py(3) rings in their optimized geometries form a π-π stacking interaction with one *C^N* ligand, similar to their crystal structures; the py(3)…Ph(*C^N*) distance of the optimized geometry of **1** (3.446 Å) is slightly shorter than that of the crystal structure, while those of the optimized geometries of **2**–**4** (**2**: 3.463 Å, **3**: 3.498 Å, and **4**: 3.490 Å) are slightly longer than those of crystal structures. In addition, the hydrogen bond is confirmed to be present in the optimized structure of **1** (2.387 Å) but not in that of **2**, similar to their crystal structures. These results indicated that the optimized structures of **1**–**4** in the S_0_ state reproduced their crystal structures well. In the T_1_ state, the iridium centers in **1**–**4** commonly form a pseudo-octahedral coordination geometry similar to that in the S_0_ state. The bonds in the first coordination sphere of the iridium ion that mostly vary between the S_0_ and T_1_ states are Ir(1)-N(4)(py2); the bond lengths in the T_1_ state are 0.06–0.11 Å shorter than those in the S_0_ state. The other bond lengths around the iridium centers between the S_0_ and T_1_ states differed by less than 0.05 Å. The py(3)…Ph(*C^N*) distances of the optimized geometries of **1**–**4** at the T_1_ state are 3.450, 3.474, 3.606, and 3.570 Å, respectively, which are slightly longer than those at the S_0_ state. These results indicate that the large structural differences between the S_0_ and T_1_ states were not observed in the optimized geometries of **1**–**4**, similar to other cyclometalated iridium complexes [21].

**Figure 2:**
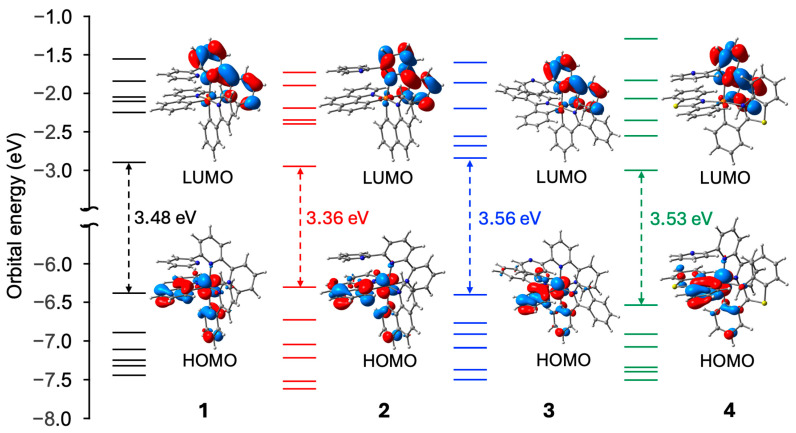
Molecular orbital diagrams (HOMO-5~LUMO+5) and HOMO/LUMO pictures of **1**–**4**.

Figure 2 shows selected molecular orbital (MO) diagrams with the highest occupied MOs (HOMOs) and lowest unoccupied MOs (LUMOs) of **1**–**4**. The MOs from HOMO-5 to LUMO+5 of **1**–**4** are shown in Figures S13–S16. The HOMOs of **1**–**4** were mainly localized at the iridium center and the two phenyl moieties in the *C^N* ligands. In **1**–**3**, HOMO-1, which is 0.52, 0.42, and 0.35 eV, respectively, more stable than their HOMOs, are localized on the two *C^N* moieties, whereas HOMO-2–HOMO-5 contain the *d*(Ir) and π(*C^N*) orbital characteristics. On the other hand, the HOMO-1–HOMO-5 of **4** consists of contributions from the *d*(Ir) and π(*C^N*) orbitals. The MO contribution from the tpy ligands in **1**–**4** was not observed in the region from HOMO to HOMO-5. In contrast, the LUMOs of **1**–**4** were predominantly localized on the py(1)-py(2) moieties of the tpy ligand, and their orbital energies were slightly different because of their structural distortions. The HOMO-LUMO gaps of **1**–**4** were estimated to be 3.48, 3.36, 3.56, and 3.53 eV, respectively. Both the LUMO+1 and LUMO+2 of **2**–**4** were distributed on the two *C^N* moieties, while those of **1** were found at the py(2)-py(3)/ppy moiety. The stabilization of MO localized on the py(3) ring in **1** is attributed to the hydrogen bonding between CH_(*ppy*)_ and N(5)_[py(3)]_.

### 2.4. Photophysical Properties

**Figure 3:**
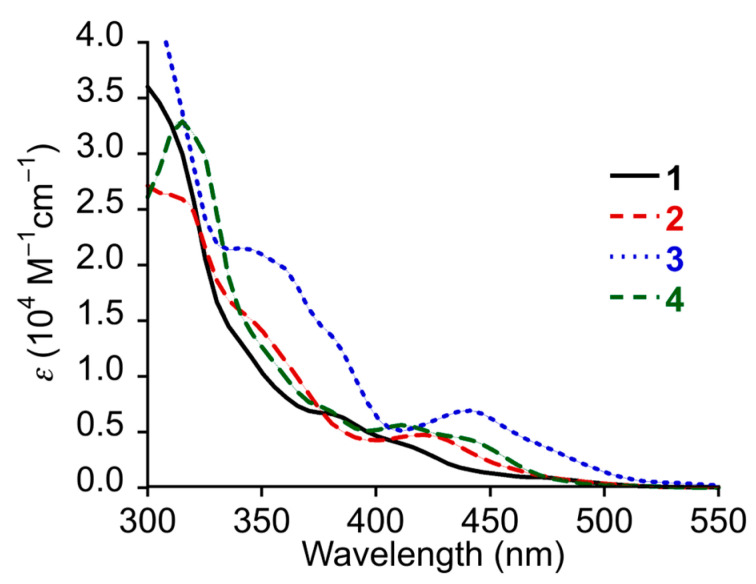
Absorption spectra of **1**–**4** in CHCl_3_.

The photophysical data for complexes **1**–**4** discussed in this study are summarized in Table 2. As shown in Figure 3, the absorption spectra of **1**–**4** in CHCl_3_ possess low-lying (shoulder) bands in the visible region and intense bands in the UV region, similar to those of other conventional cyclometalated iridium complexes. For example, **1** shows a shoulder band around 474 nm, which is assigned as the *d*(Ir)/π(ppy) (HOMO) → π*(tpy) (LUMO) transition, i.e., singlet metal–ligand to ligand charge transfer (^1^MLL′CT) by a time-dependent DFT (TDDFT) calculation (Table S5). The molar absorption coefficients (ε) of the visible absorptions of **2**–**4** are larger than those of **1** due to the increasing aromatic character of the *C^N* ligands. The visible absorption band of **2** appeared at 420 nm (ε = 4740 M^−1^cm^−1^), which is theoretically assigned as ^1^MLL′CT from *d*(Ir)/π(bzq) (HOMO) to π*(tpy) (LUMO) (Table S6). The complex that absorbs most efficiently in the visible region is **3**, with an absorption maximum at 440 nm (ε = 6933 M^−1^cm^−1^). The predominant character of this band is also able to be assigned as ^1^MLL′CT, which is the *d*(Ir)/π(piq) (HOMO) → π*(tpy) (LUMO) transition (Table S7). Furthermore, **4** has two visible absorption bands at 412 (ε = 5620 M^−1^cm^−1^) and 445 nm (ε = 4043 M^−1^cm^−1^) that are characteristic of cyclometalated iridium complexes with pbt ligands, and these absorption bands can be attributed to *d*(Ir)/π(pbt) (HOMO) → π*(tpy) (LUMO) and *d*(Ir)/π(pbt) (HOMO) → π*(pbt) (LUMO+1), respectively (Table S8).

**Figure 4:**
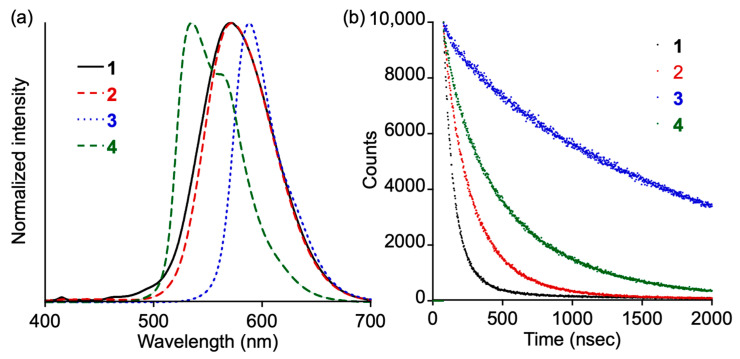
(**a**) Luminescence spectra and (**b**) luminescence decays of **1**–**4** in degassed CHCl_3_.

The luminescence spectra of **1**–**4** in degassed CHCl_3_ at 300 K are shown in Figure 4a. Furthermore, **1**–**3** possesses luminescence bands at 572, 571, and 588 nm, respectively, whereas **4** shows two continuous luminescence bands, which are well-known in the luminescence spectra of [Ir(pbt)*_2_*(*N^N*)]PF_6_ [34] at 535 and 562 nm. Since the DFT calculations confirmed that the HOMOs and LUMOs of **1**–**4** were localized on the Ir(*C^N*)_2_ and tpy moieties, respectively, the luminescence was concluded to occur from the triplet metal–ligand to ligand charge transfer (^3^MLL′CT) excited states.

To investigate the luminescent properties of **1**–**4** in detail, the luminescent lifetime (τ) and absolute luminescence quantum yield (Φ) measurements were performed in degassed CHCl_3_ at 300 K. As shown in Figure 4b, significant differences were observed in the luminescence decays between **1**–**4**. Previous reports revealed that the τ values of the [Ir(ppy)_2_(R-tpy)]PF_6_ complexes (54~125 nsec in degassed CH_2_Cl_2_) [29,30] are relatively shorter compared with the other cyclometalated iridium complexes. In fact, we confirmed that **1** exhibits a short luminescence lifetime of τ = 80 nsec, of which the value is consistent with that reported previously for **1** (68 nsec in degassed CH_2_Cl_2_) and is shorter than that of [Ir(ppy)_2_(bpy)]PF_6_ (390 nsec). On the other hand, the τ values of **2**–**4** were estimated to be 279, 1965, and 523 nsec, which are significantly longer than that of **1**. To the best of our knowledge, the τ value of **3** is the longest among the [Ir(*C^N*)(R-tpy)]PF_6_ complexes reported so far. The Φ value of **1** was determined to be 3.72%, which is about two times higher than the reported value of **1** (Φ = 1.7%) measured in degassed CH_2_Cl_2_. This result indicates that the Φ values of **1**–**4** were slightly influenced by solvent effects [35]. The Φ values of the [Ir(*C^N*)(tpy)]PF_6_ complexes were strongly enhanced by changing the *C^N* ligands, and those of **2**–**4** were 7.78, 9.57, and 7.65%, respectively. To gain further insight into the luminescent properties of **1**–**4**, the radiative rate constant (*k*_r_) and non-radiative rate constant (*k*_nr_) were estimated using Equation (1):

(1) $$\Phi =\frac{k_{r}}{k_{r}+k_{nr}}=\tau k_{r}$$

The magnitudes of the *k*_nr_ values of **1**–**4** were approximately 10 times larger than the *k*_r_ values, indicating that the transition processes from the T_1_ state to the S_0_ state in **1**–**4** were dominated by thermal deactivation rather than phosphorescence.

### 2.5. Electrochemical Properties

**Figure 5:**
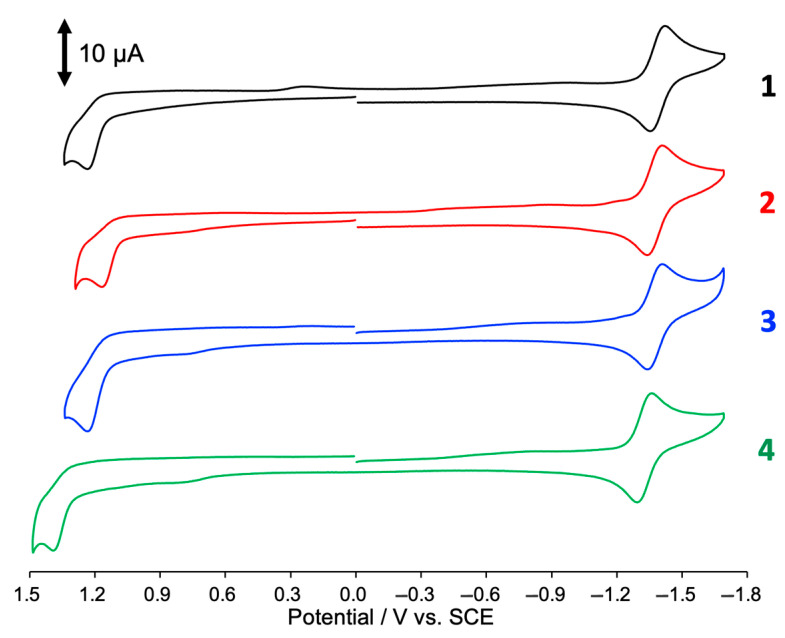
CV diagrams of **1**–**4** in degassed PC containing 0.10 M TBAPF_6_.

Finally, to clarify the electrochemical properties of **1**–**4** in degassed PC, cyclic voltammetry (CV) measurements were performed. As shown in Figure 5, the CV diagrams of **1**–**4** were similar; they underwent one reversible reduction and one irreversible oxidation. The estimated redox potentials (E_1/2_) of **1**–**4** are summarized in Table 3. The E_1/2_ values at each reduction and oxidation process of **1** were estimated as −1.38 and +1.18 V vs. SCE, which are shifted to the positive and negative directions, respectively, compared with those of [Ir(ppy)_2_(bpy)]^+^ (E_1/2_ = −1.42 V and +1.25 V vs. SCE in CH_3_CN) [10]. The results of the DFT calculations suggested that the reduction and oxidation of **1**–**4** occurred at the tpy and Ir(ppy)_2_ moieties, respectively. Indeed, the E_1/2_ values of the reduction processes of **2**–**4** were almost the same as those of **1**, and the ratios of *I*_pa_ and *I*_pc_ of **1**–**4** were approximately 1:1 (*I*_pc_/I_pa_ = 0.96 for **1**, 1.0 for **2**, 0.94 for **3**, and 1.0 for **4**). On the other hand, a significant difference was observed in the E_1/2_ values of their oxidation processes. The most easily oxidized complex was **2**, showing an E_1/2_ value at +1.12 V. On the other hand, **4** possesses the largest oxidation potential (E_1/2_ = +1.34 V vs. SCE) among the complexes investigated in this study.

## 3. Experimental Section

### 3.1. Materials and Instruments

The chloride-bridged iridium dimer complexes, [Ir(*C^N*)_2_Cl]_2_, were prepared as previously described [20,36]. 2,2′:6′,2′′-Terpyridine (tpy), 2-phenylpyridine (Hppy), benzo[*h*]quinoline (Hbzq), 1-phenylisoquinoline (Hpiq), and 2-phenylbenzothiazoline (Hpbt) were purchased from Tokyo Chemical Industry Co. Ltd. (Tokyo, Japan), and the other reagents were obtained from Fujifilm-Wako Pure Chemical Industry (Osaka, Japan).

The CHN elemental analyses were performed using a Yanaco (Tokyo, Japan) CHN corder MT-6 instrument at Shimane University. The proton nuclear magnetic resonance (^1^H NMR) spectra in DMSO-*d*_6_ were recorded on a JEOL (Tokyo, Japan) JNM-ECX500 spectrometer with the chemical shifts (δ) referenced to the residual DMSO peak (δ = 2.49 ppm). Electrospray time-of-flight ionization mass spectrometry (ESI-TOF-MS) was performed using a Bruker (Billerica, MA, USA) micrOTOF II instrument in the positive-ion mode using sodium formate as a mass calibrant. Attenuated total reflection-Fourier transform infrared (ATR-FT-IR) spectroscopy was measured using a JASCO (Tokyo, Japan) FT/IR-6300 spectrophotometer equipped with an ATR PRO ONE module. Absorption and emission spectra were recorded using the JASCO (Tokyo, Japan) V-670 and FP-8300 spectrophotometers, respectively. The emission lifetimes were estimated using a HORIBA (Kyoto, Japan) FluoroCube instrument with an excitation wavelength of 370 nm, and the absolute quantum yields were determined using a HAMAMATSU Photonics (Shidzuoka, Japan) Quantaurus-QY system with an excitation wavelength of 370 nm. Cyclic voltammetry (CV) measurements (scan rate: 100 mV/s) were performed in degassed PC solution containing 0.10 M tetrabutylammonium hexafluorophosphate (TBAPF_6_) using a HOKUTO DENKO HZ-7000 system (Tokyo, Japan) equipped with a glassy carbon disk (3.0 mm diameter), platinum wire, and saturated calomel electrode (SCE), which were employed as the working, counter, and reference electrodes, respectively.

### 3.2. Synthesis of [Ir(ppy)_2_(tpy)]PF_6_ (***1***)

[Ir(ppy)_2_Cl]_2_ (53.6 mg, 0.0500 mmol) and tpy (58.4 mg, 0.110 mmol) were dissolved in 30.0 mL of 1,2-dichloroethane/methanol (1:1) solution and refluxed for 24 h. After the resultant solution was evaporated, the residue was collected on a membrane filter and washed with diethyl ether. The yellow powder obtained was dissolved in H_2_O, followed by the addition of NH_4_PF_6_ (489.2 mg, 3.00 mmol). A yellow precipitate was collected, washed with H_2_O, and dried under vacuum at 393 K. Yield: 70.8 mg (0.0806 mmol, 80.6%). Anal. Calc. for C_37_H_27_N_5_Ir_1_P_1_F_6_: C, 50.57; H, 3.10; N, 7.97%. Found: C, 50.31; H, 3.22; N, 7.78%. ^1^H NMR (500 MHz, DMSO-*d_6_*): δ = 8.87 (t, 2H), 8.78 (broad s, 1H), 8.30 (m, 2H), 8.19 (d, 1H), 8.13 (broad d, 1H), 8.08 (d, 1H), 7.95 (td, 1H), 7.89 (td, 1H), 7.79 (dd, 1H), 7.61 (m, 2H), 7.58 (dd, 1H), 7.50 (d, 1H), 7.45 (d, 1H), 7.28 (m, 1H), 7.18 (td, 1H), 7.08 (m, 1H), 6.96 (m, 1H), 6.90 (td, 1H), 6.72 (td, 1H), 6.67 (broad d, 1H), 6.53 (td, 1H), 6.23 (td, 1H), 5.76 (dd, 1H) 5.32 (d, 1H) ppm. ESI-TOF-MS: calc. for [M]^+^: 734.1892 *m*/*z*; found 734.1890 *m*/*z*.

### 3.3. Synthesis of [Ir(bzq)_2_(tpy)]PF_6_ (***2***)

A preparation method similar to that used for **1** was applied for the synthesis of **2**, but [Ir(bzq)_2_Cl]_2_ (58.4 mg, 0.0500 mmol) was used instead of [Ir(ppy)_2_Cl]_2_. Yield: 83.1 mg (0.0897 mmol, 89.7%). Anal. Calc. for C_41_H_27_N_5_Ir_1_P_1_F_6_: C, 53.13; H, 2.94; N, 7.56%. Found: C, 52.84; H, 3.23; N, 7.59%. ^1^H NMR (500 MHz, DMSO-*d_6_*): δ = 8.95 (dd, 1H), 8.92 (d, 1H), 8.72 (broad s, 1H), 8.57 (qd, 2H), 8.36 (t, 1H), 8.25 (td, 1H), 8.07 (d, 1H), 7.86 (q, 3H), 7.79 (q, 3H), 7.72 (q, 1H), 7.61 (d, 1H), 7.57 (q, 1H), 7.54 (dd, 1H), 7.50 (m, 1H), 7.41 (d, 1H), 7.05 (dd, 1H), 6.96 (t, 1H), 6.72 (broad d, 2H), 6.50 (t, 1H), 5.65 (dd, 1H), 5.30 (d, 1H) ppm. ESI-TOF-MS: calc. for [M]^+^: 782.1893 *m*/*z*; found 782.1890 *m*/*z*.

### 3.4. Synthesis of [Ir(piq)_2_(tpy)]PF_6_ (***3***)

A preparation method similar to that used for **1** was applied for the synthesis of **3**, but [Ir(piq)_2_Cl]_2_ (63.6 mg, 0.0500 mmol) was used instead of [Ir(ppy)_2_Cl]_2_. Yield: 80.0 mg (0.0817 mmol, 81.7%). Anal. Calc. for C_45_H_31_N_5_Ir_1_P_1_F_6_: C, 55.21; H, 3.19; N, 7.15%. Found: C, 55.00; H, 3.31; N, 7.23%. ^1^H NMR (500 MHz, DMSO-*d_6_*): δ = 8.98 (d, 1H), 8.89 (d, 1H), 8.84 (m, 3H), 8.30 (d, 1H), 8.27 (dd, 1H), 8.23 (t, 1H), 8.15 (dd, 1H), 8.08 (d, 1H), 8.04 (m, 1H), 7.90 (m, 4H), 7.83 (d, 1H), 7.72 (d, 1H), 7.58 (m, 1H), 7.51 (dd, 1H), 7.48 (d, 1H), 7.45 (dd, 1H), 7.38 (d, 1H), 7.26 (td, 1H), 7.04 (m, 1H), 6.90 (qd, 1H), 6.81 (broad s, 1H), 6.76 (td, 1H), 6.62 (m, 1H), 6.25 (td, 1H), 6.12(dd, 1H), 5.45 (dd, 1H) ppm. ESI-TOF-MS: calc. for [M]^+^: 834.2206 *m*/*z*; found 834.2198 *m*/*z*.

### 3.5. Synthesis of [Ir(pbt)_2_(tpy)]PF_6_ (***4***)

A preparation method similar to that used for **1** was applied for the synthesis of **4**, but [Ir(pbt)_2_Cl]_2_ (65.0 mg, 0.0500 mmol) was used instead of [Ir(ppy)_2_Cl]_2_. Yield: 90.7 mg (0.0915 mmol, 91.5%). Anal. Calc. for C_41_H_27_N_5_S_2_Ir_1_P_1_F_6_: C, 49.69; H, 2.75; N, 7.07%. Found: C, 49.41; H, 2.92; N, 7.10%. ^1^H NMR (500 MHz, DMSO-*d_6_*): δ = 8.86 (td, 2H), 8.38 (t, 1H), 8.25 (m, 3H), 7.99 (d, 1H), 7.83 (dd, 1H), 7.71 (dd, 1H), 7.65 (m, 2H), 7.49 (td, 1H), 7.42 (td, 1H), 7.35 (m, 2H), 7.15 (m, 2H), 7.01 (td, 1H), 6.90 (m, 2H), 6.83 (m, 2H),6.61 (td, 1H), 6.47 (td, 1H), 6.19 (d, 1H), 5.91 (d, 1H), 5.83 (d, 1H) ppm. ESI-TOF-MS: calc. for [M]^+^: 846.1331 *m*/*z*; found 846.1347 *m*/*z*.

### 3.6. Crystallography

The diffraction data of **1** and **3** were collected at 100 K using a RIGAKU (Tokyo, Japan) XtaLAB Synergy system equipped with a Hypix-6000 detector and a molybdenum rotating anode X-ray source, whereas those of **2** and **4** were collected at 100 K using a Pilatus3 X 1M CdTe detector and monochromated synchrotron radiation (X-ray energy: 30.0 keV) installed at the BL02B1 beamline of the SPring-8 synchrotron radiation facility. The collected diffraction data were processed and reduced using a CrysAlisPro program (version 1.171.42.94a) [37]. The initial models of the structures were obtained using the SHELXT-2014 program [38] with the intrinsic phasing method and were then refined with full-matrix least squares on *F*^2^ using the SHELXL-2019 program [39] in the Olex2 software (version 1.5) [40]. Non-hydrogen atoms were refined anisotropically, and hydrogen atoms were fixed at the calculated positions and refined as riding models. The electron densities of the disordered solvents in **2** and **4** were removed using the solvent mask routine of Olex2 software. The crystallographic data for the final refined structures of **1**–**4** are summarized in Table 4, and the structural parameters of **1**–**4** are summarized in Tables S9–S12. These crystallographic data can be obtained free of charge from the Cambridge Crystallographic Data Centre (CCDC); deposition numbers **1**–**4** are CCDC 2407543–2407546, respectively. 

### 3.7. Density Functional Theory (DFT) Calculations

The density functional theory (DFT) calculations applied in this study were performed using the dispersion-corrected hybrid functional PBE1PBE-D3BJ [41,42] in combination with the SDD basis set [43] for the iridium atom and the cc-pVDZ basis set [44] for the other atoms. Geometry optimizations at singlet (S_0_) and triplet (T_1_) states were performed without symmetry constraints, and the resulting optimized geometries were characterized using vibrational frequency analyses (no imaginary frequency). Time-dependent DFT (TDDFT) calculations [45] were performed to obtain the excitation energies (wavelengths) and their oscillator strengths and assignments of excitation characters. All the calculations were performed using the Gaussian 16 (Revision C.01) software package [46].

## 4. Conclusions

The synthesis, characterization, electronic structures, and photophysical and electrochemical properties of a series of bis-cyclometalated iridium complexes with the tpy ligand, [Ir(*C^N*)_2_(tpy)]PF_6_ (**1**–**4**), were systematically investigated by using both experimental and theoretical approaches. The crystal structures of all complexes **1**–**4** were determined by SCXRD analyses; the intramolecular π-π stacking interactions between the pendant pyridyl group of the tpy ligand and the phenyl moiety of the *C^N* ligand were observed in all complexes **1**–**4**, whereas an intramolecular hydrogen bonding interaction between them was only found in the structure of **1**. The *C^N* ligands significantly influenced not only the molecular structure but also the photophysical properties of the [Ir(*C^N*)_2_(tpy)]PF_6_ complexes. The Φ and τ values of **2**–**4** were enhanced compared with those of previously reported **1**. To the best of our knowledge, the τ value of **3** is the longest among the [Ir(*C^N*)(R-tpy)]PF_6_ complexes reported so far. The further improvement of Φ and τ values and the photochemical applications of the [Ir(*C^N*)_2_(tpy)]PF_6_ derivatives are currently in progress in our group.

## Figures and Tables

**Table 1:** Selected distances of supramolecular interactions between py(3)/N(5) and *C^N* ligands and rotation angles between two pyridyl rings in crystal structures of **1**–**4**.

Complex	py(3)…Ph[*C^N*] Distance (Å) *	N(5)…HC[*C^N*] Distance (Å)	py(1)-py(2) Rotation Angle (°)	py(2)-py(3) Rotation Angle (°)
**1**	3.486	2.571	26.09	48.21
**2**	3.381	4.176	17.32	67.76
**3**	3.410	―	16.12	59.32
**4**	3.389	―	4.50	71.52

* Centroid—centroid distance.

**Table 2:** Photophysical properties of **1**–**4** in CHCl_3_.

Complex	Absorption (nm) [ε (M^−1^cm^−1^)]	Luminescence (nm)	τ (nsec)	Φ (%)	*k*_r_ (10^5^ s^−1^)	*k*_nr_ (10^6^ s^−1^)
**1**	309^sh^[33139], 382^sh^[6551], 415^sh^[3707], 474^sh^[913]	572	80	3.72	4.66	12.1
**2**	315^sh^[25938], 348^sh^[14583], 420[4740]	571	279	7.78	2.79	3.31
**3**	343[21536], 360^sh^[19700], 380^sh^[13811], 440[6933]	588	1965	9.57	0.487	0.460
**4**	314[32929], 380^sh^[6927], 412[5620], 445^sh^[4043]	535, 562	523	7.65	1.46	1.77

^sh^ shoulder band.

**Table 3:** Redox potentials and peak separations (ΔE) of **1**–**4** in degassed PC containing 0.10 M TBAPF_6_.

Complex	Reduction [V vs. SCE] (ΔE [mV])	Oxidation [V vs. SCE]
**1**	−1.38 (67)	+1.18^ir^
**2**	−1.37 (67)	+1.12^ir^
**3**	−1.37 (67)	+1.17^ir^
**4**	−1.33 (66)	+1.34^ir^

^ir^ Irreversible wave.

**Table 4:** Crystallographic data of **1**–**4**.

	1	2	3	4
Radiation type	Mo Kα	synchrotron	Mo Kα	synchrotron
Radiation Wavelength	0.71073	0.4108	0.71073	0.4108
Chemical formula	C_43_H_39_F_6_IrN_5_O_2_P	C_41_H_27_F_6_IrN_5_P	C_46_H_35_F_6_IrN_5_OP	C_55_H_43_F_6_IrN_5_PS_2_
Formula weight	994.96	926.84	1010.96	1175.23
Crystal system	Monoclinic	Orthorhombic	Monoclinic	Monoclinic
Space group	*P* 1 2_1_/*c* 1	*Pnma*	*P* 1 2_1_/*n* 1	*P* 1 2_1_/*c* 1
*a* (Å)	17.8340 (5)	18.40720 (10)	11.5148 (8)	11.70630 (10)
*b* (Å)	26.3098 (8)	21.81460 (10)	28.9068 (19)	23.1619 (2)
*c* (Å)	8.2954 (3)	19.57810 (10)	11.8870 (9)	18.49030 (10)
α (deg)	90	90	90	90
β (deg)	91.468 (3)	90	110.105 (8)	90.7350 (10)
γ (deg)	90	90	90	90
*V* (Å^3^)	3891.0 (2)	7861.50 (7)	3715.6 (5)	5013.05 (7)
*Z*	4	8	4	4
*D*_calc_(g cm^−3^)	1.698	1.566	1.807	1.557
μ (mm^−1^)	3.547	0.872	3.714	0.709
*F*(000)	1976	3632	2000	2344
*R*_1_ (*I* > 2*σ(I)*)	0.0253	0.0243	0.0395	0.0324
*wR*_2_ (*I* > 2*σ(I)*)	0.0553	0.0550	0.0830	0.0772
*R*_1_ (all data)	0.0326	0.0248	0.0626	0.0330
*wR*_2_ (all data)	0.0571	0.0553	0.0903	0.0775
GOF on *F*^2^	1.080	1.059	1.029	1.208

## Data Availability

All data in this study are included in the manuscript and Supplementary Materials.

## Supplementary Materials

The following supporting information can be downloaded at: https://www.mdpi.com/article/10.3390/molecules30010193/s1, Figure S1: Observed and simulated ESI-TOF-MS spectra of **1**; Figure S2: Observed and simulated ESI-TOF-MS spectra of **2**; Figure S3: Observed and simulated ESI-TOF-MS spectra of **3**; Figure S4: Observed and simulated ESI-TOF-MS spectra of **4**; Figure S5: ^1^H NMR spectrum of **1** in DMSO-*d*_6_; Figure S6: ^1^H NMR spectrum of **2** in DMSO-*d*_6_; Figure S7: ^1^H NMR spectrum of **3** in DMSO-*d*_6_; Figure S8: ^1^H NMR spectrum of **4** in DMSO-*d*_6_; Figure S9: Optimized structures of **1** at S_0_ and T_1_ states; Figure S10: Optimized structures of **2** at S_0_ and T_1_ states; Figure S11: Optimized structures of **3** at S_0_ and T_1_ states; Figure S12: Optimized structures of **4** at S_0_ and T_1_ states; Figure S13: MO pictures from HOMO-5 to LUMO+5 of **1**; Figure S14: MO pictures from HOMO-5 to LUMO+5 of **2**; Figure S15: MO pictures from HOMO-5 to LUMO+5 of **3**; Figure S16: MO pictures from HOMO-5 to LUMO+5 of **4**; Figure S17: Comparison between experimental spectra (black line) and calculated excitations (vertical red line) of (a) **1**, (b) **2**, (c) **3**, and (d) **4**; Table S1: Structural parameters of primary coordination sphere in crystal and DFT-optimized geometries of **1**; Table S2: Structural parameters of primary coordination sphere in crystal and DFT-optimized geometries of **2**; Table S3: Structural parameters of primary coordination sphere in crystal and DFT-optimized geometries of **3**; Table S4: Structural parameters of primary coordination sphere in crystal and DFT-optimized geometries of **4**; Table S5: Results of TDDFT calculation of **1** (H and L indicate the HOMO and LUMO, respectively); Table S6: Results of TDDFT calculation of **2** (H and L indicate the HOMO and LUMO, respectively); Table S7: Results of TDDFT calculation of **3** (H and L indicate the HOMO and LUMO, respectively); Table S8: Results of TDDFT calculation of **4** (H and L indicate the HOMO and LUMO, respectively); Table S9: Structural parameters of crystal structure of **1** (bond lengths: Å, bond angles: °); Table S10: Structural parameters of crystal structure of **2** (bond lengths: Å, bond angles: °); Table S11: Structural parameters of crystal structure of **3** (bond lengths: Å, bond angles: °); Table S12: Structural parameters of crystal structure of **4** (bond lengths: Å, bond angles: °).
