# Supplementary material for: Luminescent Iridium–Terpyridine Complexes with Various Bis-Cyclometalated Ligands
Source: Molecules. 2025 Jan 6;30(1):193. doi: 10.3390/molecules30010193 (PMC11722081; doi:10.3390/molecules30010193)
Supplement: Supplementary file 1 [file molecules-30-00193-s001.zip › molecules-3381163-supplementary.pdf]

Supplementary materials

Luminescent Iridium–Terpyridine Complexes with Various Bis-Cyclometalated Ligands

Ko Ikeda, Natsumi Yano,* Makoto Handa, and Yusuke Kataoka*

Contents

Figure S1. Observed and simulated ESI-TOF-MS spectra of **1**.

Figure S2. Observed and simulated ESI-TOF-MS spectra of **2**.

Figure S3. Observed and simulated ESI-TOF-MS spectra of **3**.

Figure S4. Observed and simulated ESI-TOF-MS spectra of **4**.

Figure S5. ^{1}H NMR spectrum of **1** in DMSO-d_6.

Figure S6. ^{1}H NMR spectrum of **2** in DMSO-d_6.

Figure S7. ^{1}H NMR spectrum of **3** in DMSO-d_6.

Figure S8. ^{1}H NMR spectrum of **4** in DMSO-d_6.

Figure S9. Optimized structures of **1** at S_0 and T_1 states.

Figure S10. Optimized structures of **2** at S_0 and T_1 states.

Figure S11. Optimized structures of **3** at S_0 and T_1 states.

Figure S12. Optimized structures of **4** at S_0 and T_1 states.

Figure S13. MO pictures from HOMO-5 to LUMO+5 of **1**.

Figure S14. MO pictures from HOMO-5 to LUMO+5 of **2**.

Figure S15. MO pictures from HOMO-5 to LUMO+5 of **3**.

Figure S16. MO pictures from HOMO-5 to LUMO+5 of **4**.

Figure S17. Comparison between experimental spectra (black line) and calculated excitations (vertical red line) of (a) **1**, (b) **2**, (c) **3**, and (d) **4**.

Table S1. Structural parameters of primary coordination sphere in crystal and DFT-optimized geometries of **1**.

Table S2. Structural parameters of primary coordination sphere in crystal and DFT-optimized geometries of **2**.

Table S3. Structural parameters of primary coordination sphere in crystal and DFT-optimized geometries of **3**.

Table S4. Structural parameters of primary coordination sphere in crystal and DFT-optimized geometries of **4**.

Table S5. Results of TDDFT calculation of **1** (H and L indicate the HOMO and LUMO, respectively).

Table S6. Results of TDDFT calculation of **2** (H and L indicate the HOMO and LUMO, respectively).

Table S7. Results of TDDFT calculation of **3** (H and L indicate the HOMO and LUMO, respectively).

Table S8. Results of TDDFT calculation of **4** (H and L indicate the HOMO and LUMO, respectively).

Table S9. Structural parameters of crystal structure of **1** (bond lengths: Å, bond angles: °).

Table S10. Structural parameters of crystal structure of **2** (bond lengths: Å, bond angles: °).

Table S11. Structural parameters of crystal structure of **3** (bond lengths: Å, bond angles: °).

Table S12. Structural parameters of crystal structure of **4** (bond lengths: Å, bond angles: °).

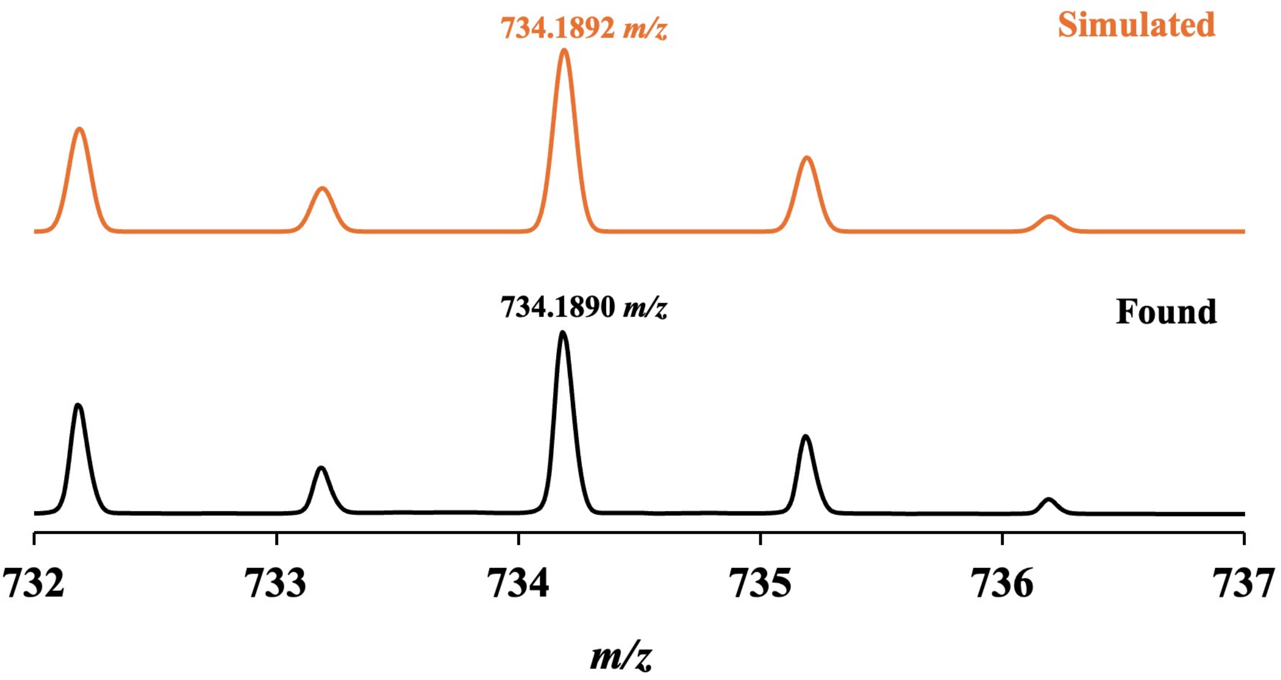

Figure S1. Observed and simulated ESI-TOF-MS spectra of **1**.

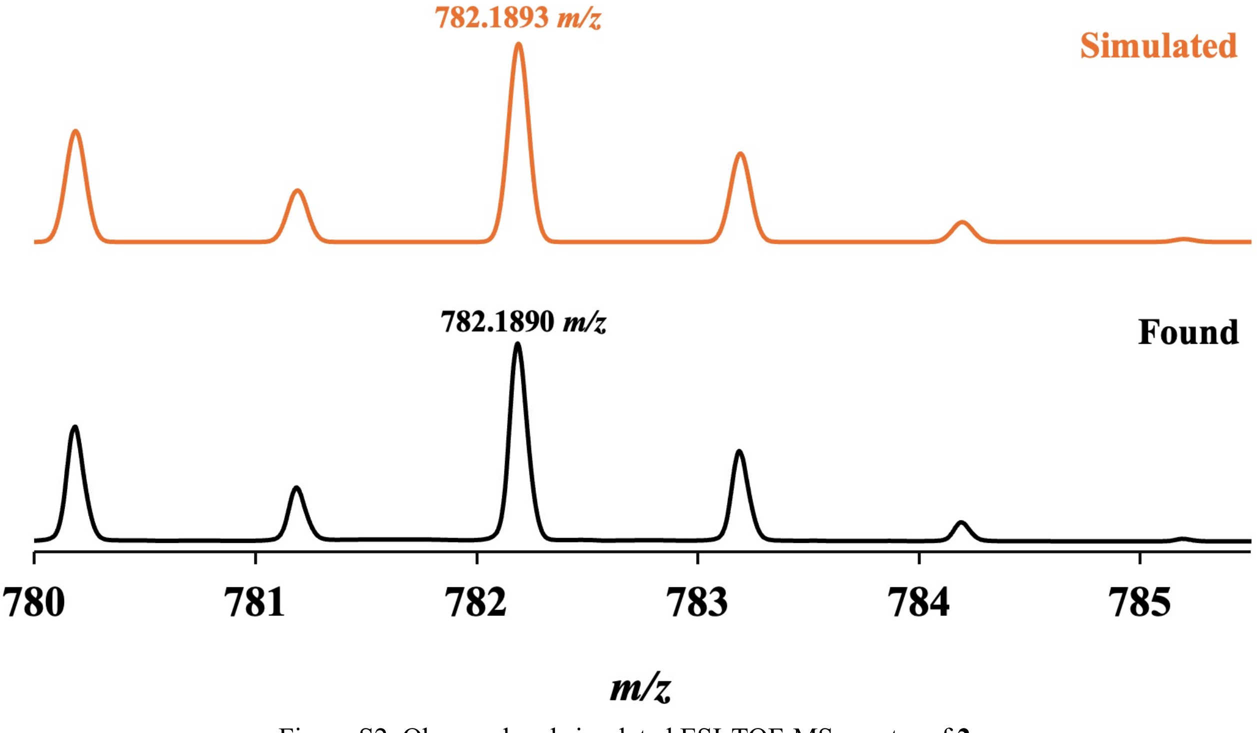

Figure S2. Observed and simulated ESI-TOF-MS spectra of **2**.

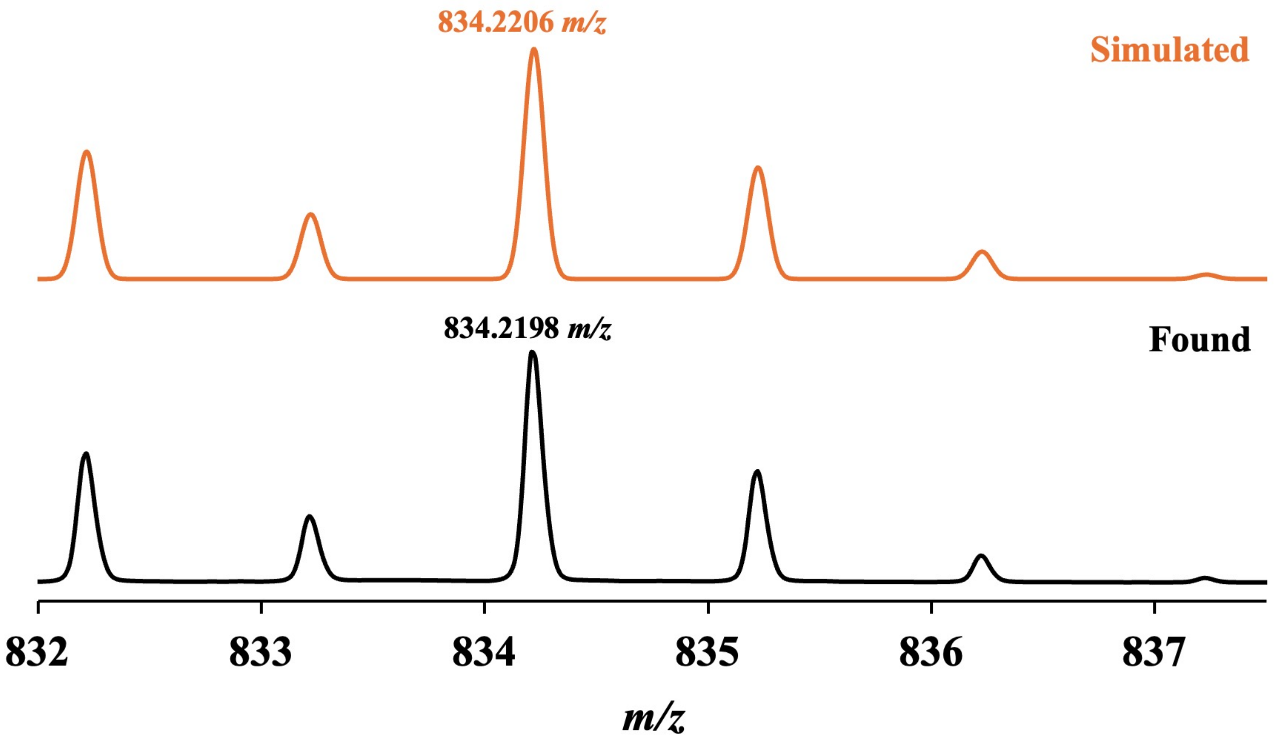

Figure S3. Observed and simulated ESI-TOF-MS spectra of **3**.

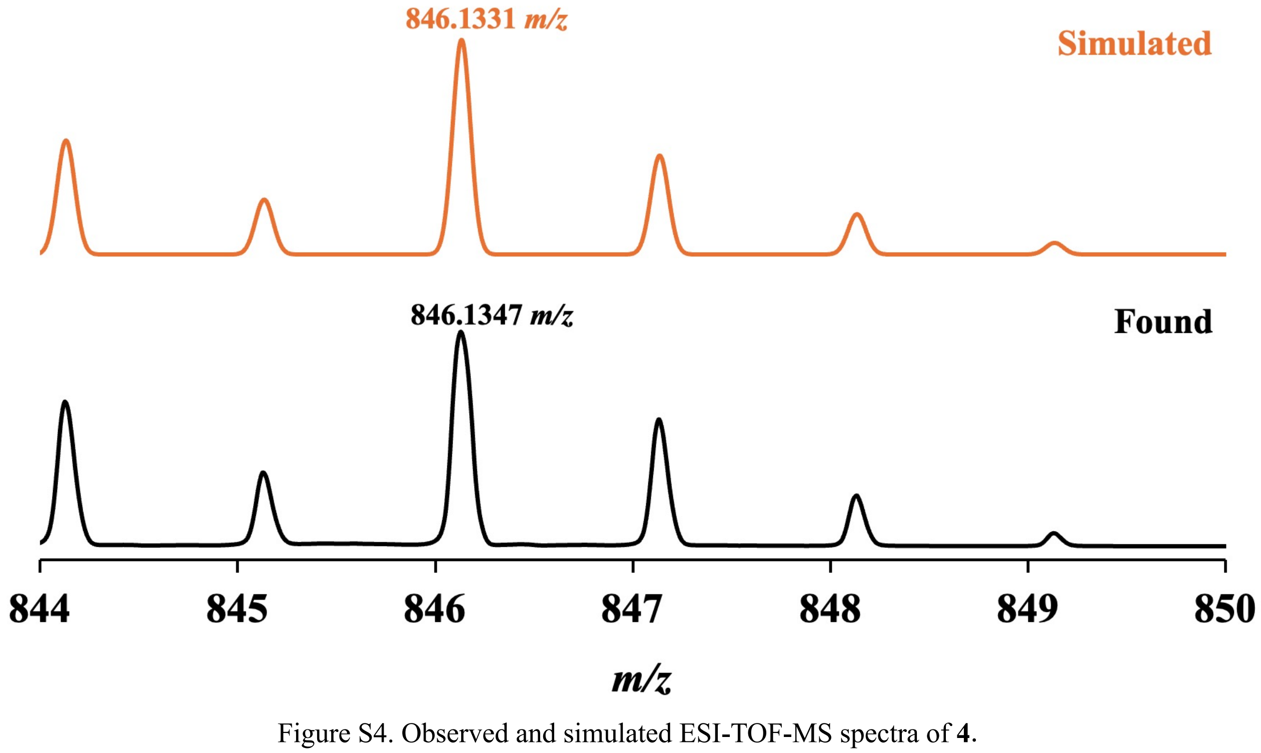

Figure S4. Observed and simulated ESI-TOF-MS spectra of **4**.

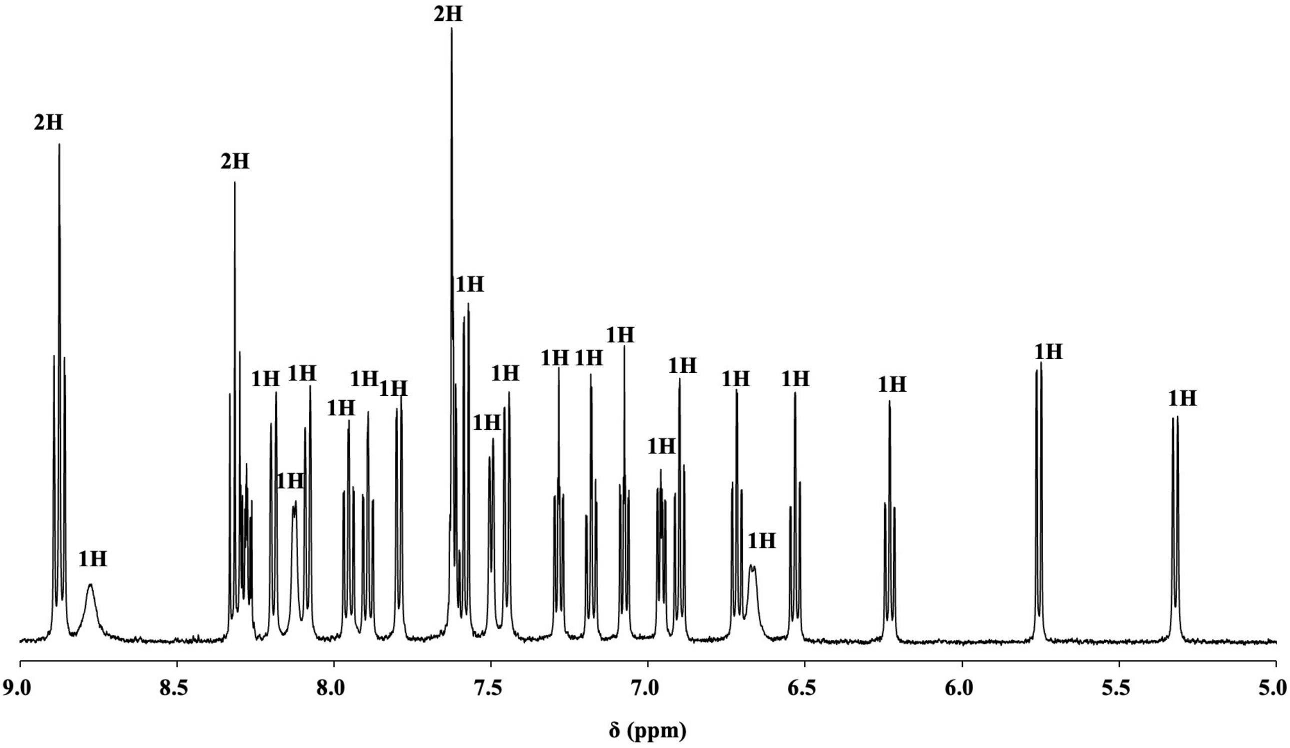

Figure S5. ^{1}H NMR spectrum of **1** in DMSO-d_6.

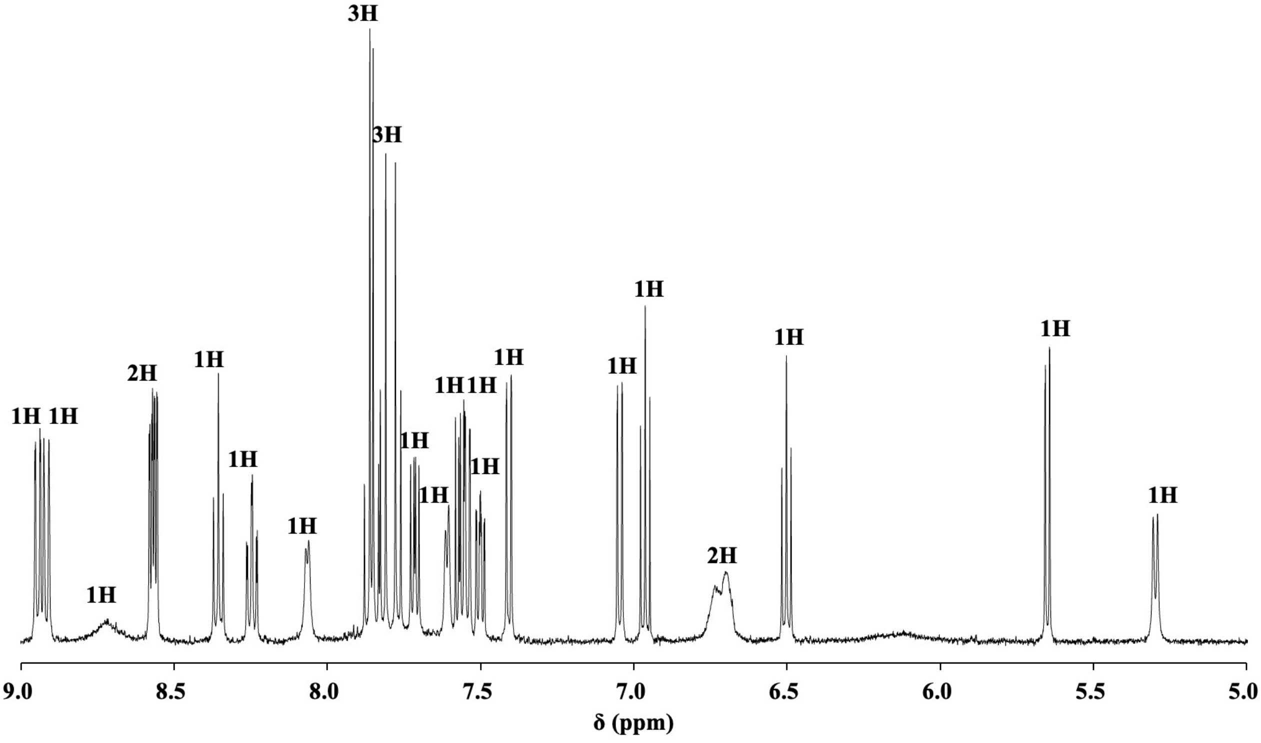

Figure S6. ^{1}H NMR spectrum of **2** in DMSO-d_6.

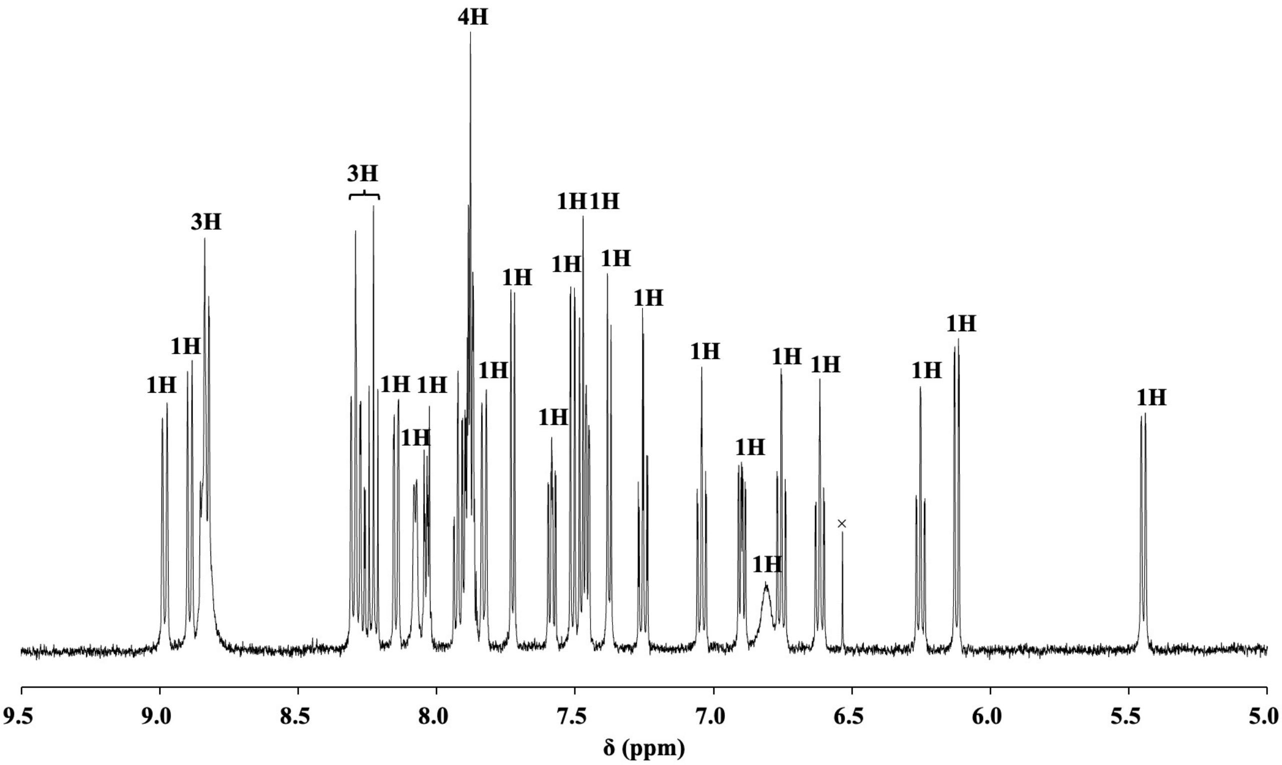

Figure S7. ^{1}H NMR spectrum of **3** in DMSO-d_6.

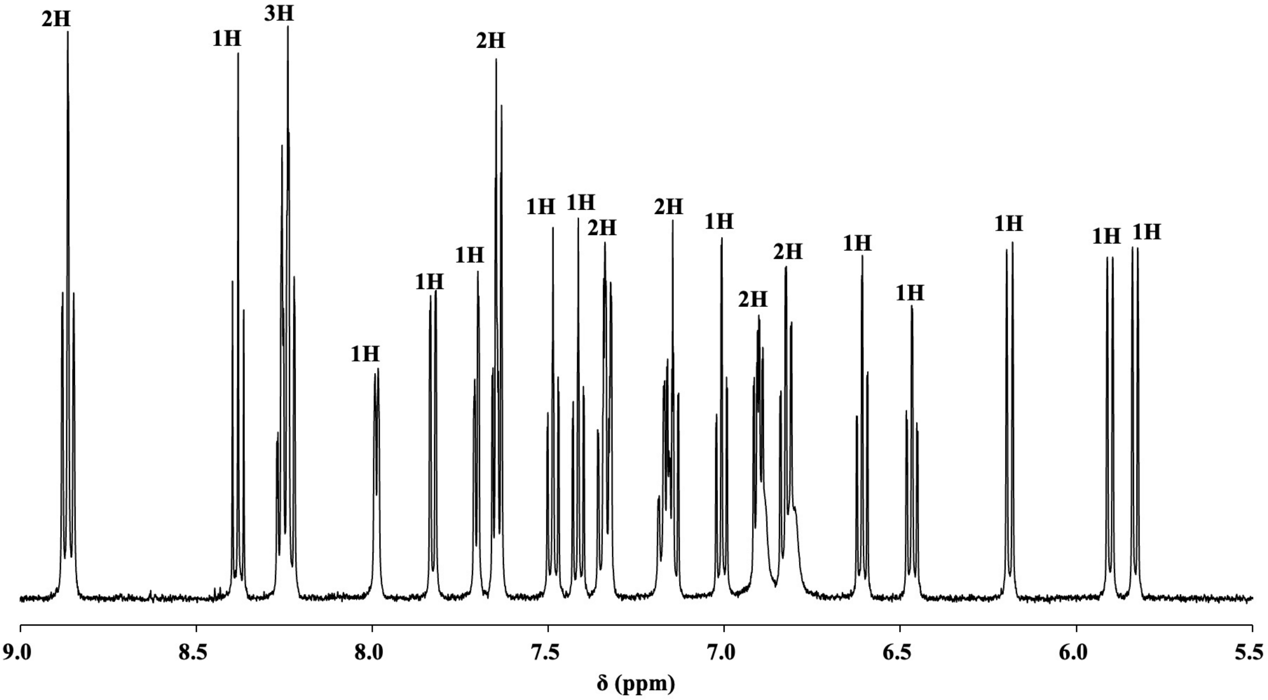

Figure S8. ^{1}H NMR spectrum of **4** in DMSO-d_6.

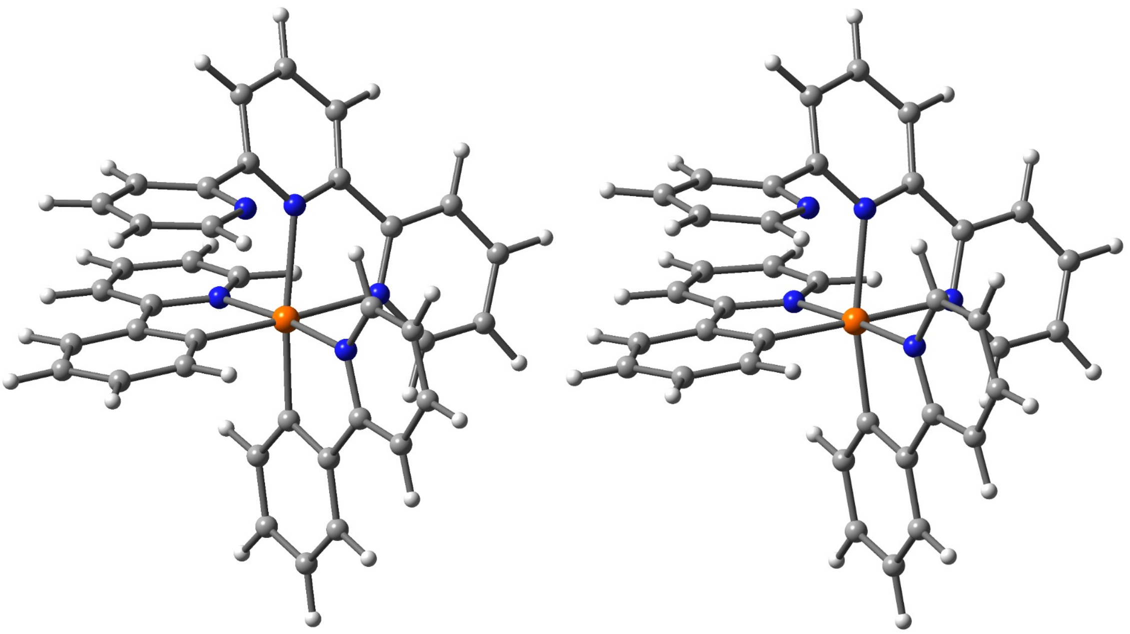

S_0 state **T_1 state**

Figure S9. Optimized structures of **1** at S_0 and T_1 states.

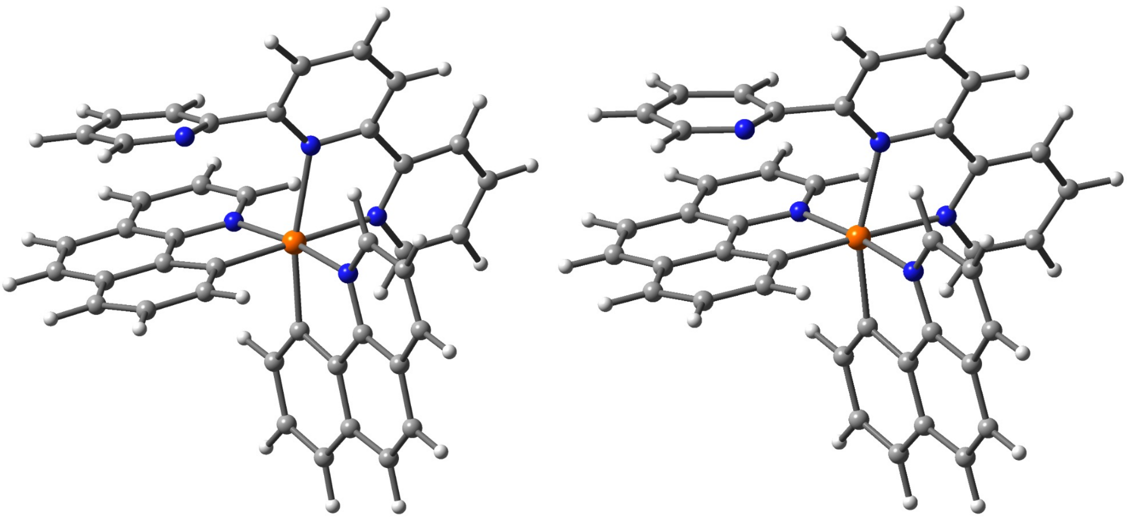

S_0 state **T_1 state**

Figure S10. Optimized structures of **2** at S_0 and T_1 states.

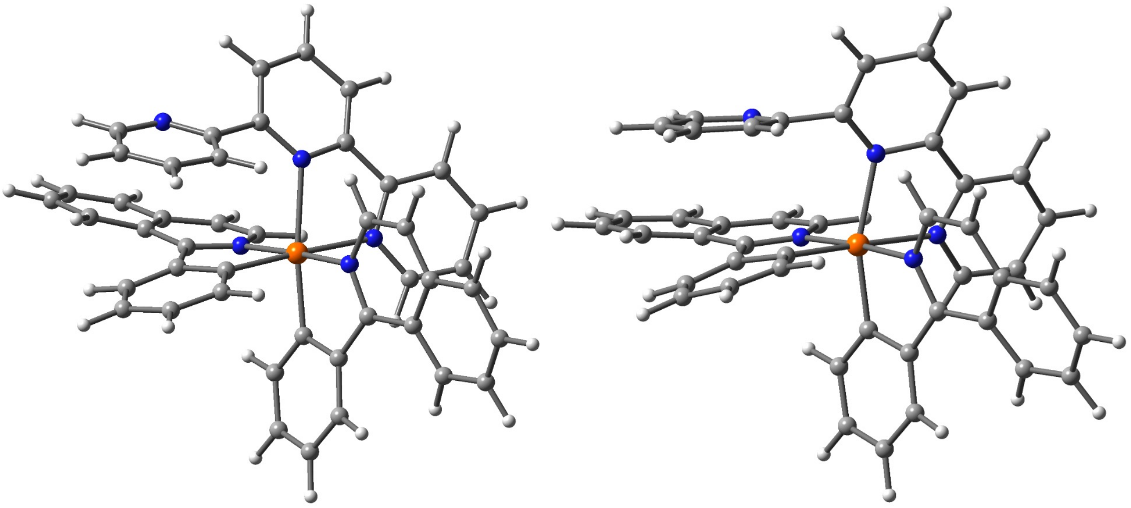

S_0 state **T_1 state**

Figure S11. Optimized structures of **3** at S_0 and T_1 states.

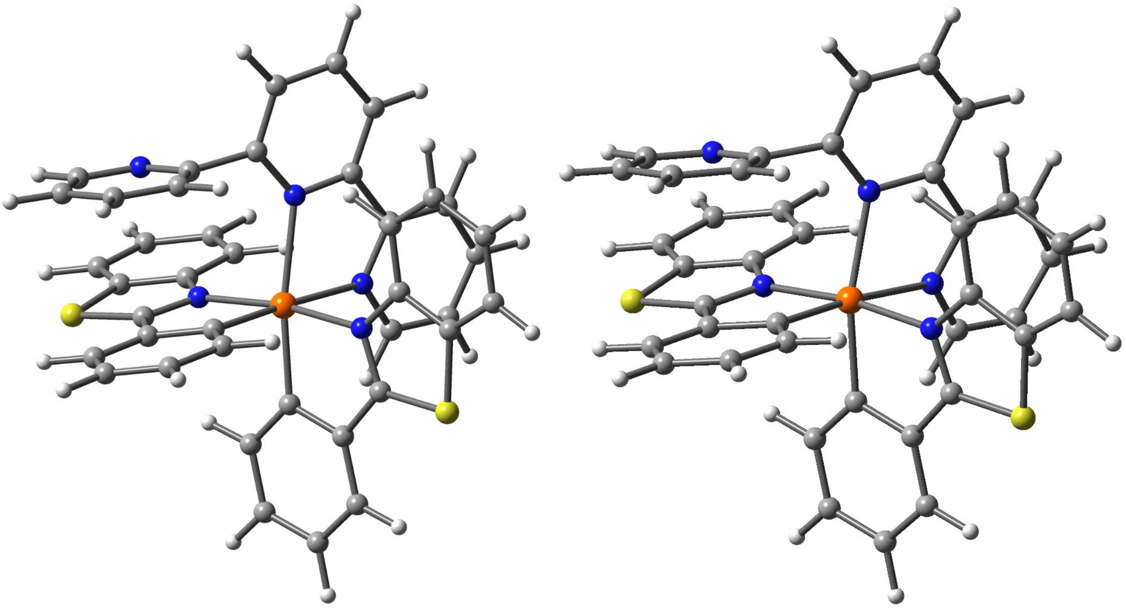

S_0 state **T_1 state**

Figure S12. Optimized structures of **4** at S_0 and T_1 states.

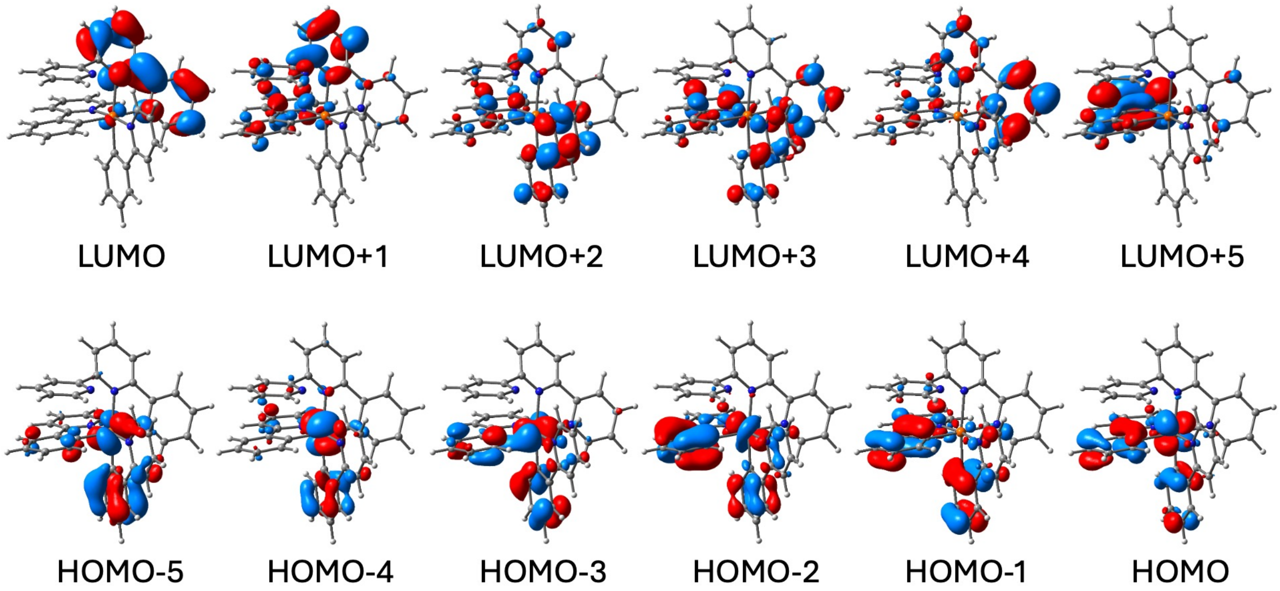

Figure S13. MO pictures from HOMO-5 to LUMO+5 of **1**.

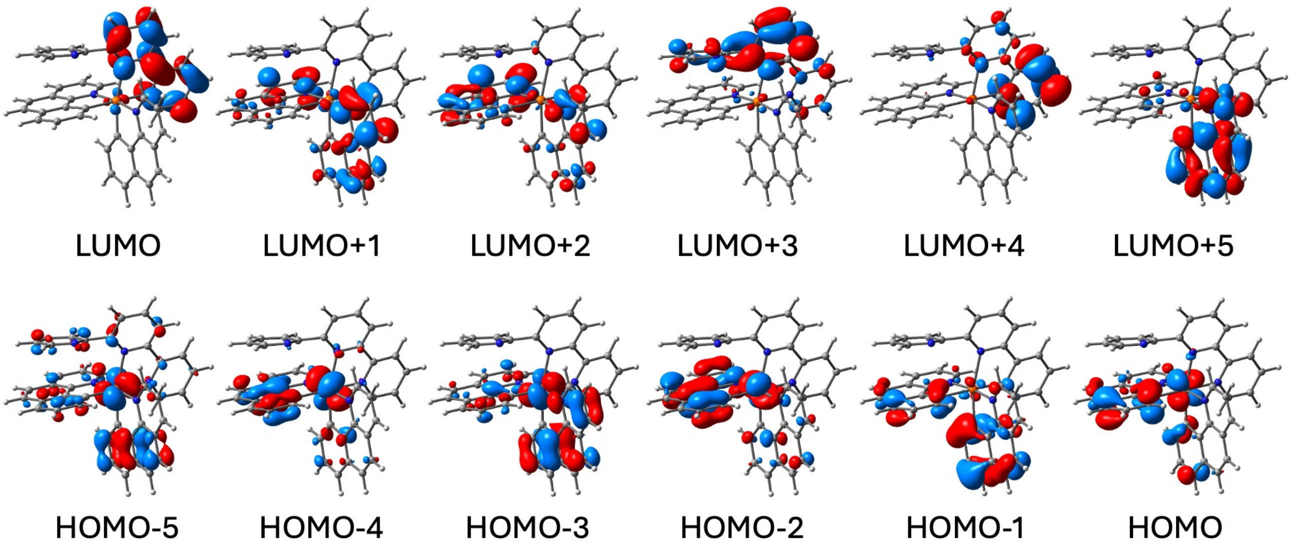

Figure S14. MO pictures from HOMO-5 to LUMO+5 of **2**.

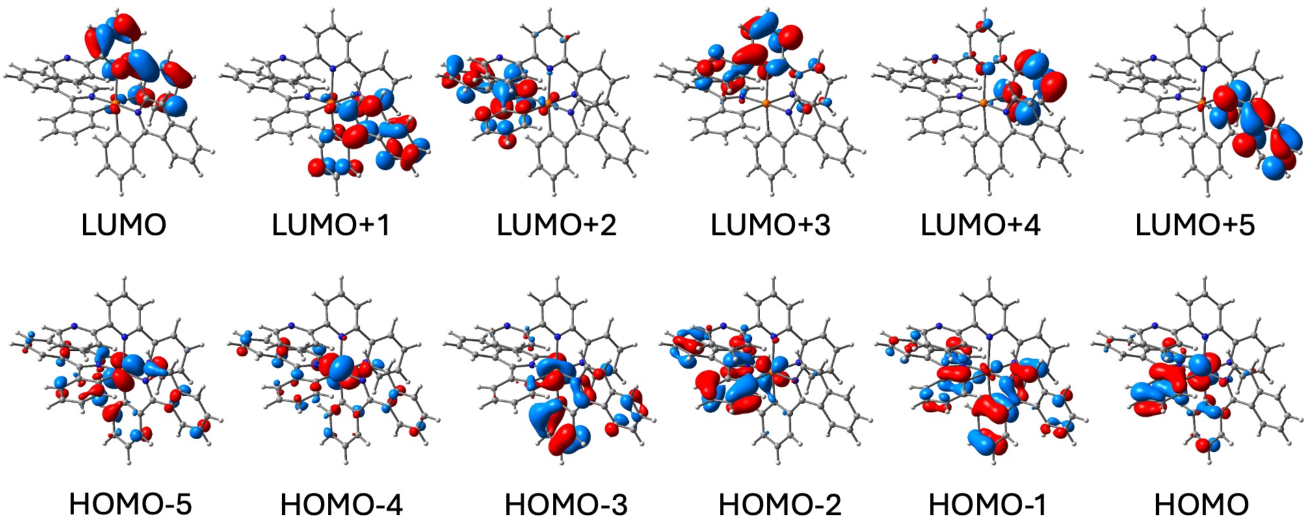

Figure S15. MO pictures from HOMO-5 to LUMO+5 of **3**.

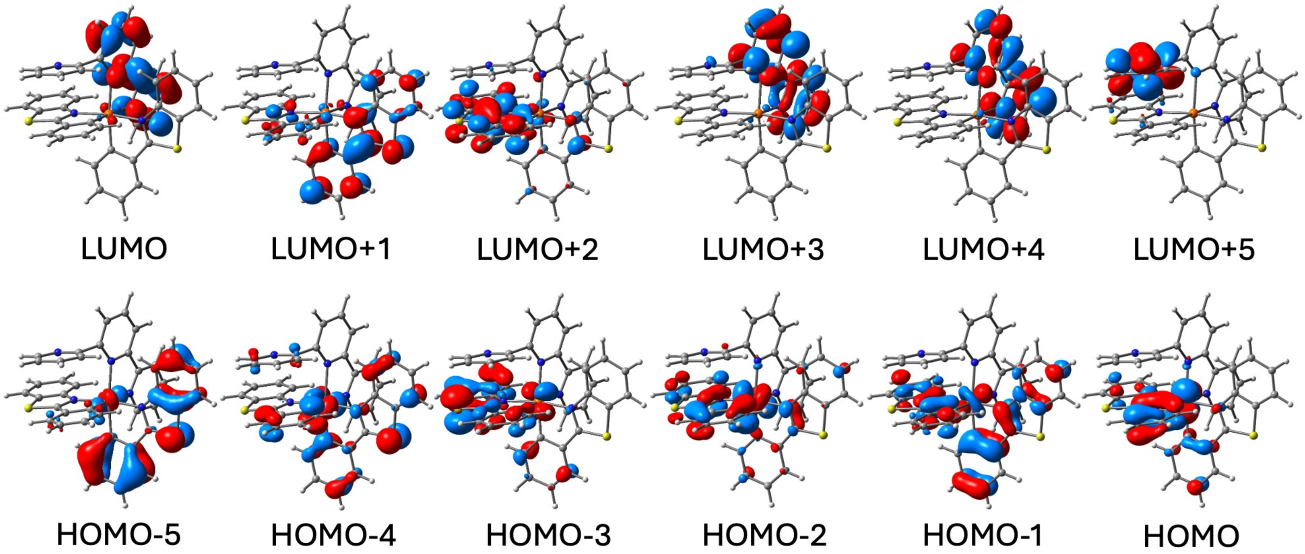

Figure S16. MO pictures from HOMO-5 to LUMO+5 of **4**.

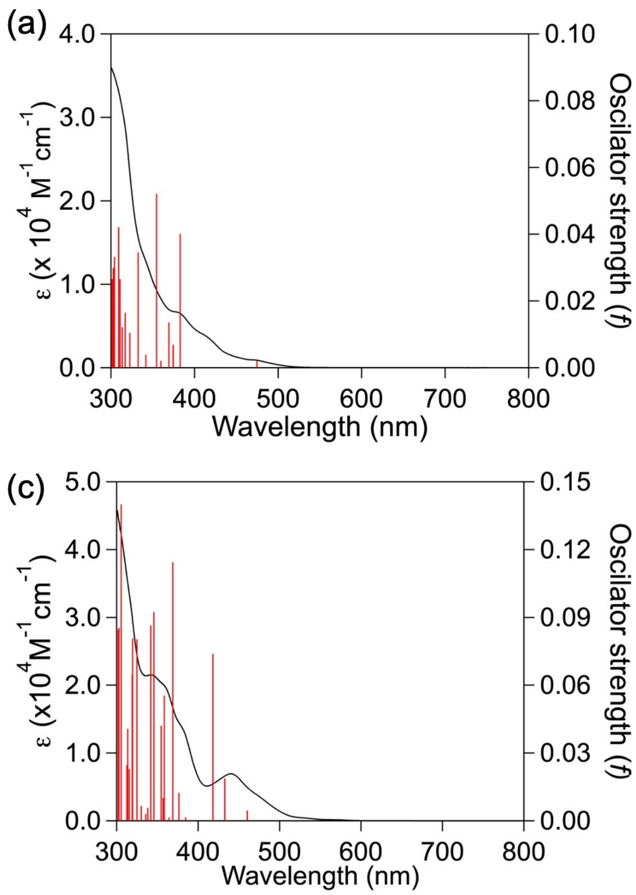

Figure S17. Comparison between experimental spectra (black line) and calculated excitations (vertical red line) of (a) **1**, (b) **2**, (c) **3**, and (d) **4**.

Table S1. Structural parameters of primary coordination sphere in crystal and DFT-optimized geometries of **1**.

	crystal structure	optimized geometry at S_0 state	optimized geometry at T_1 state
Bond lengths (Å)			
Ir(1)-C(1)	1.999(2)	1.998	2.000
Ir(1)-N(1)	2.035(2)	2.053	2.057
Ir(1)-C(12)	2.018(2)	2.011	1.975
Ir(1)-N(2)	2.052(2)	2.062	2.062
Ir(1)-N(3)	2.1411(19)	2.157	2.168
Ir(1)-N(4)	2.2238(19)	2.235	2.180
Bond angles (°)			
C(1)-Ir(1)-N(1)	80.26(9)	80.54	80.81
C(12)-Ir(1)-N(2)	80.12(9)	80.13	80.95
N(3)-Ir(1)-N(4)	75.63(7)	75.22	75.94

Table S2. Structural parameters of primary coordination sphere in crystal and DFT-optimized geometries of **2**.

	crystal structure	optimized geometry at S_0 state	optimized geometry at T_1 state
Bond lengths (Å)			
Ir(1)-C(1)	2.003(2)	2.005	2.015
Ir(1)-N(1)	2.058(2)	2.073	2.066
Ir(1)-C(14)	2.022(2)	2.017	1.969
Ir(1)-N(2)	2.0362(18)	2.056	2.061
Ir(1)-N(3)	2.1286(19)	2.158	2.177
Ir(1)-N(4)	2.199(2)	2.228	2.157
Bond angles (°)			
C(1)-Ir(1)-N(1)	81.32(9)	81.33	81.92
C(14)-Ir(1)-N(2)	81.44(9)	81.23	81.93
N(3)-Ir(1)-N(4)	76.14(8)	75.03	75.16

Table S3. Structural parameters of primary coordination sphere in crystal and DFT-optimized geometries of **3**.

	crystal structure	optimized geometry at S_0 state	optimized geometry at T_1 state
Bond lengths (Å)			
Ir(1)-C(1)	1.999(5)	1.990	1.999
Ir(1)-N(1)	2.044(3)	2.053	2.061
Ir(1)-C(16)	2.008(4)	2.006	1.961
Ir(1)-N(2)	2.037(3)	2.055	2.048
Ir(1)-N(3)	2.126(3)	2.165	2.182
Ir(1)-N(4)	2.246(4)	2.272	2.159
Bond angles (°)			
C(1)-Ir(1)-N(1)	79.81(16)	79.81	80.80
C(16)-Ir(1)-N(2)	79.31(14)	79.40	80.54
N(3)-Ir(1)-N(4)	75.71(14)	75.29	75.40

Table S4. Structural parameters of primary coordination sphere in crystal and DFT-optimized geometries of **4**.

	crystal structure	optimized geometry at S_0 state	optimized geometry at T_1 state
Bond lengths (Å)			
Ir(1)-C(1)	2.008(3)	2.009	2.023
Ir(1)-N(1)	2.065(3)	2.083	2.072
Ir(1)-C(14)	2.016(3)	2.015	1.973
Ir(1)-N(2)	2.043(3)	2.059	2.054
Ir(1)-N(3)	2.127(3)	2.161	2.187
Ir(1)-N(4)	2.202(3)	2.214	2.137
Bond angles (°)			
C(1)-Ir(1)-N(1)	79.91(13)	80.09	81.03
C(14)-Ir(1)-N(2)	79.89(12)	79.85	80.63
N(3)-Ir(1)-N(4)	75.97(11)	75.23	75.57

Table S5. Results of TDDFT calculation of **1** (H and L indicate the HOMO and LUMO, respectively).

S_n	Wavelength (nm)	*f*	Major contributions
1	474.5	0.0021	H→L (97%)
2	382.7	0.0401	H→L+1 (71%), H→L+2 (18%)
3	374.5	0.0067	H-1→L (52%), H-2→L (21%), H-4→L (15%)
4	374.3	0.0069	H-1→L (42%), H-2→L (27%), H-4→L (18%)
5	369.2	0.0136	H→L+2 (63%), H→L+3 (20%), H→L+1 (10%)
6	359.7	0.0021	H→L+3 (58%), H→L+1 (11%), H-3→L (10%)
7	354.5	0.0521	H-3→L (44%), H-5→L (16%), H-4→L (16%), H→L+3 (13%)
8	341.7	0.0039	H-2→L (48%), H-4→L (39%), H-3→L (10%)
9	332.3	0.0346	H→L+4 (91%)
10	322.5	0.0105	H-5→L (73%), H-3→L (17%)
11	316.9	0.0165	H-1→L+1 (83%)
12	313.4	0.0122	H→L+5 (89%)
13	310.7	0.0266	H-2→L+1 (40%), H-3→L+1 (16%), H-2→L+2 (11%), H-4→L+1 (10%)
14	309.1	0.0421	H-1→L+2 (71%), H-1→L+3 (10%)
15	304.0	0.0332	H→L+6 (52%), H-1→L+3 (24%)
16	302.7	0.0299	H-2→L+2 (20%), H-1→L+3 (15%), H-4→L+2 (13%), H→L+6 (12%)
17	302.2	0.0036	H-3→L+1 (21%), H→L+6 (18%), H-2→L+1 (16%), H-3→L+3 (10%)
18	301.4	0.0266	H-1→L+3 (31%), H-2→L+2 (15%), H-4→L+2 (13%)

Table S6. Results of TDDFT calculation of **2** (H and L indicate the HOMO and LUMO, respectively).

S_n	Wavelength (nm)	*f*	Major contributions
1	483.3	0.0042	H→L (97%)
2	403.8	0.0626	H→L+1 (91%)
3	397.5	0.0009	H-1→L (89%)
4	394.3	0.0102	H→L+2 (84%)
5	381	0.0024	H-2→L (79%), H-4→L (15%)
6	370	0.0015	H→L+3 (93%)
7	359.3	0.0764	H-3→L (81%), H-5→L (10%)
8	348.7	0.026	H-1→L+1 (86%)
9	341.1	0.0165	H→L+4 (60%), H-1→L+2 (26%)
10	340.1	0.0439	H-1→L+2 (61%), H→L+4 (22%)
11	334.2	0.0113	H→L+5 (34%), H→L+6 (23%), H-2→L+2 (15%), H→L+4 (10%)
12	331.2	0.0171	H→L+5 (33%), H-2→L+1 (22%), H→L+6 (16%), H-4→L (10%)
13	328.3	0.0036	H-4→L (68%), H-2→L (14%)
14	323.5	0.1152	H-2→L+1 (51%), H→L+6 (26%)
15	318.0	0.0053	H-1→L+3 (50%), H-5→L (16%), H-2→L+2 (14%)
16	317.2	0.0214	H-1→L+3 (35%), H-5→L (24%), H-2→L+2 (20%)
17	316.4	0.0277	H-5→L (38%), H-2→L+2 (23%), H-3→L+1 (19%)
18	310.8	0.071	H-3→L+2 (71%)
19	309.2	0.0266	H-3→L+1 (44%), H→L+5 (12%), H-2→L+2 (10%)
20	305.6	0.0809	H-6→L (62%), H-2→L+3 (13%)
21	302.7	0.085	H-2→L+3 (49%), H-7→L (21%)
22	300.2	0.0438	H-7→L (47%), H-6→L (16%), H-2→L+3 (11%), H-1→L+4 (11%)

Table S7. Results of TDDFT calculation of **3** (H and L indicate the HOMO and LUMO, respectively).

S_n	Wavelength (nm)	*f*	Major contributions
1	460.36014	0.0046	H→L (92%)
2	432.709291	0.0188	H→L+1 (91%)
3	418.046372	0.0739	H→L+2 (95%)
4	384.459031	0.0016	H-1→L (93%)
5	376.371177	0.0124	H-2→L (77%), H-4→L (13%)
6	368.890786	0.1145	H-1→L+1 (90%)
7	364.198787	0.0015	H→L+3 (59%), H-3→L (32%)
8	358.356532	0.0554	H-3→L (39%), H→L+3 (26%), H-2→L+1 (22%)
9	356.932845	0.0101	H-2→L+1 (56%), H-3→L (10%)
10	354.524171	0.0421	H-1→L+2 (90%)
11	345.552377	0.0924	H-2→L+2 (76%), H-3→L+1 (15%)
12	341.714282	0.0864	H-3→L+1 (72%), H-2→L+2 (15%)
13	338.089532	0.0057	H-4→L (69%), H-2→L (10%)
14	335.636689	0.0031	H-3→L+2 (75%)
15	330.087572	0.0066	H→L+4 (87%)
16	324.821045	0.0803	H-4→L+1 (62%)
17	319.612789	0.0807	H-1→L+3 (34%), H-4→L+2 (27%), H-2→L+3 (15%)
18	319.061718	0.0645	H-5→L (73%)
19	315.4172	0.0229	H-1→L+3 (31%), H-4→L+2 (17%), H-5→L+1 (11%), H-5→L (10%)
20	313.423816	0.0408	H-2→L+3 (36%), H-5→L+1 (22%), H-4→L+2 (14%)
21	312.460164	0.0247	H-2→L+3 (40%), H-5→L+1 (26%), H-1→L+3 (17%)
22	305.432447	0.1400	H→L+5 (75%), H-5→L+1 (13%)

Table S8. Results of TDDFT calculation of **4** (H and L indicate the HOMO and LUMO, respectively).

S_n	Wavelength (nm)	*f*	Major contributions
1	460.1	0.0023	H→L (97%)
2	405.4	0.1009	H→L+1 (94%)
3	393.3	0.0181	H-1→L (91%)
4	384.3	0.0076	H-2→L (79%), H→L+2 (12%)
5	380.2	0.0380	H→L+2 (81%), H-2→L (10%)
6	349.5	0.0659	H-1→L+1 (89%)
7	343.9	0.0572	H-3→L (58%), H-4→L (29%)
8	338.5	0.0273	H-2→L+1 (73%)
9	337.5	0.0037	H→L+3 (64%), H-4→L (11%), H-2→L+1 (11%)
10	335.6	0.0033	H-4→L (36%), H→L+3 (29%), H-3→L (23%)
11	330.4	0.1290	H-1→L+2 (84%)
12	324.6	0.0027	H-5→L (85%)
13	319.7	0.0792	H-2→L+2 (65%), H→L+4 (20%)
14	318.3	0.0275	H-2→L+2 (16%), H→L+4 (70%)
15	310.5	0.0282	H-4→L+1 (45%), H-3→L+1 (36%)

Table S9. Structural parameters of crystal structure of **1** (bond lengths: Å, bond angles: °).

bond length (Å)							
Ir1-N2	2.052(2)	C1-C6	1.410(3)	C14-C13	1.388(3)	C16-C15	1.379(3)
Ir1-N1	2.035(2)	C1-C2	1.396(3)	C14-C15	1.383(4)	C30-C31	1.377(4)
Ir1-N4	2.2238(19)	C17-C18	1.460(3)	C27-C28	1.481(3)	C36-C37	1.381(4)
Ir1-N3	2.1411(19)	C17-C12	1.407(3)	C27-C26	1.385(3)	C36-C35	1.375(4)
Ir1-C1	1.999(2)	C17-C16	1.397(3)	C24-C23	1.378(3)	P1-F3	1.5918(18)
Ir1-C12	2.018(2)	C18-C19	1.390(3)	C24-C25	1.382(4)	P1-F5	1.5872(19)
N2-C18	1.370(3)	C12-C13	1.401(3)	C3-C2	1.385(3)	P1-F1	1.5915(17)
N2-C22	1.346(3)	C6-C7	1.466(3)	C3-C4	1.385(4)	P1-F6	1.5770(19)
N1-C7	1.362(3)	C6-C5	1.399(3)	C21-C20	1.389(4)	P1-F4	1.576(2)
N1-C11	1.356(3)	C7-C8	1.387(3)	C21-C22	1.370(4)	P1-F2	1.573(2)
N4-C32	1.353(3)	C11-C10	1.372(3)	C20-C19	1.380(3)	O2-C41	1.209(3)
N4-C28	1.353(3)	C9-C8	1.373(4)	C33-C34	1.389(3)	O1-C38	1.209(3)
N3-C27	1.356(3)	C9-C10	1.394(3)	C34-C35	1.388(4)	C41-C43	1.496(4)
N3-C23	1.341(3)	C5-C4	1.380(4)	C28-C29	1.387(3)	C41-C42	1.500(4)
N5-C33	1.336(3)	C32-C33	1.485(3)	C25-C26	1.385(3)	C38-C39	1.476(4)
N5-C37	1.335(3)	C32-C31	1.393(3)	C29-C30	1.383(4)	C38-C40	1.496(4)
bond angles (°)							
N2-Ir1-N4	80.64(7)	F2-P1-F1	89.28(13)	C13-C12-C17	117.1(2)	C34-C33-C32	121.4(2)
N2-Ir1-N3	97.47(8)	F2-P1-F6	91.47(14)	C10C6-C7	115.0(2)	C35-C34-C33	118.3(3)
N1-Ir1-N2	176.48(7)	F2-P1-F4	178.23(14)	C5-C6-C1	121.3(2)	N2-C22-C21	122.8(2)
N1-Ir1-N4	101.99(7)	C18-N2-Ir1	115.49(15)	C5-C6-C7	123.8(2)	N4-C28-C27	115.5(2)
N1-Ir1-N3	85.51(7)	C22-N2-Ir1	124.95(17)	N1-C7-C6	113.3(2)	N4-C28-C29	122.6(2)
N3-Ir1-N4	75.63(7)	C22-N2-C18	119.1(2)	N1-C7-C8	120.5(2)	C29-C28-C27	121.9(2)
C1-Ir1-N2	97.38(9)	C7-N1-Ir1	116.55(16)	C8-C7-C6	126.2(2)	N3-C23-C24	122.3(2)
C1-Ir1-N1	80.26(9)	C11-N1-Ir1	123.85(15)	N1-C11-C10	122.0(2)	C24-C25-C26	119.2(2)
C1-Ir1-N4	173.30(8)	C11-N1-C7	119.4(2)	C8-C9-C10	119.7(2)	C3-C2-C1	121.0(2)
C1-Ir1-N3	98.37(8)	C32-N4-Ir1	126.22(16)	C4-C5-C6	119.8(2)	C30-C29-C28	118.6(2)
C1-Ir1-C12	84.30(9)	C28-N4-Ir1	110.56(15)	N4-C32-C33	118.6(2)	C27-C26-C25	118.8(2)
C12-Ir1-N2	80.12(9)	C28-N4-C32	118.2(2)	N4-C32-C31	121.1(2)	C15-C16-C17	119.5(2)
C12-Ir1-N1	96.98(9)	C27-N3-Ir1	116.16(15)	C31-C32-C33	120.1(2)	C31-C30-C29	119.1(2)
C12-Ir1-N4	101.59(8)	C23-N3-Ir1	124.99(16)	C15-C14-C13	120.9(2)	C20-C19-C18	120.1(2)
C12-Ir1-N3	176.65(8)	C23-N3-C27	118.6(2)	C9-C8-C7	119.7(2)	C30-C31-C32	119.9(2)
F5-P1-F3	178.64(11)	C37-N5-C33	116.9(2)	N3-C27-C28	115.0(2)	C35-C36-C37	118.8(3)
F5-P1-F1	88.71(11)	C6-C1-Ir1	114.88(17)	N3-C27-C26	121.9(2)	N5-C37-C36	123.7(3)
F1-P1-F3	89.93(10)	C2-C1-Ir1	127.75(18)	C26-C27-C28	123.0(2)	O2-C41-C43	122.2(3)
F6-P1-F3	89.01(11)	C2-C1-C6	117.3(2)	C23-C24-C25	119.2(2)	O2-C41-C42	121.7(3)
F6-P1-F5	92.35(11)	C12-C17-C18	115.3(2)	C11-C10-C9	118.6(2)	C43-C41-C42	116.1(3)
F6-P1-F1	178.67(14)	C16-C17-C18	122.9(2)	C2-C3-C4	121.0(2)	C16-C15-C14	119.8(2)
F4-P1-F3	90.65(12)	C16-C17-C12	121.8(2)	C22-C21-C20	118.7(2)	C5-C4-C3	119.6(2)
F4-P1-F5	89.31(13)	N2-C18-C17	113.9(2)	C14-C13-C12	120.8(2)	C36-C35-C34	118.7(3)
F4-P1-F1	89.87(13)	N2-C18-C19	119.9(2)	C19-C20-C21	119.2(2)	O1-C38-C39	121.1(3)
F4-P1-F6	89.34(13)	C19-C18-C17	126.1(2)	N5-C33-C32	114.9(2)	O1-C38-C40	122.8(3)
F2-P1-F3	87.79(12)	C17-C12-Ir1	114.61(16)	N5-C33-C34	123.5(2)	C39-C38-C40	116.1(3)
F2-P1-F5	92.22(13)	C13-C12-Ir1	128.16(18)				

Table S10. Structural parameters of crystal structure of **2** (bond lengths: Å, bond angles: °).

bond length (Å)							
Ir1-N2	2.0362(18)	C19-C18	1.412(3)	C38-C37	1.353(4)	C28-C29	1.368(5)
Ir1-N1	2.058(2)	C1-C2	1.378(4)	C38-C39	1.389(4)	N5-C41	1.357(5)
Ir1-N3	2.1286(19)	C1-C6	1.419(3)	C32-C33	1.390(4)	C30-C29	1.371(5)
Ir1-N4	2.199(2)	C7-C6	1.409(4)	C15-C16	1.407(4)	C3-C4	1.375(4)
Ir1-C14	2.022(2)	C7-C8	1.411(3)	C12-C5	1.436(4)	C35-C34	1.367(5)
Ir1-C14	2.003(2)	C24-C23	1.392(3)	C12-C13	1.349(5)	C39-C40	1.366(5)
N2-C24	1.329(3)	C20-C21	1.406(3)	C18-C17	1.397(4)	C40-C41	1.339(6)
N2-C20	1.368(3)	C2-C3	1.412(4)	C18-C25	1.431(4)	P1-F5	1.5973(14)
N1-C7	1.373(3)	C31-C32	1.464(4)	C23-C22	1.369(4)	P1-F2	1.588(3)
N1-C11	1.331(3)	C31-C30	1.385(4)	C5-C4	1.402(4)	P1-F3	1.584(2)
N3-C31	1.349(3)	C6-C5	1.408(4)	C37-N5	1.345(4)	P1-F4	1.588(3)
N3-C27	1.338(4)	C11-C10	1.393(3)	C26-C21	1.438(4)	P1-F1	1.552(3)
N4-C36	1.352(3)	C27-C28	1.384(4)	C26-C25	1.347(5)	P2-F8	1.590(3)
N4-C32	1.357(3)	C8-C13	1.432(4)	C21-C22	1.392(4)	P2-F10	1.596(2)
C14-C19	1.415(3)	C8-C9	1.400(4)	C17-C16	1.371(4)	P2-F7	1.618(4)
C14-C15	1.385(3)	C36-C37	1.491(4)	C10-C9	1.365(4)	P2-F6	1.578(3)
C19-C20	1.413(4)	C36-C35	1.386(4)	C33-C34	1.369(5)	P2-F9	1.551(4)
bond angles (°)							
N2-Ir1-N1	171.00(8)	F6-P2-F8	176.6(2)	C6-C7-C8	122.0(2)	C6-C5-C12	117.9(3)
N2-Ir1-N3	95.84(8)	F6-F2-F10	90.02(9)	N2-C24-C23	122.0(2)	C4-C5-C6	117.2(2)
N2-Ir1-N4	92.28(7)	F6-P2-F7	88.5(2)	N2-C20-C19	116.2(2)	C4-C5-C12	124.9(3)
N1-Ir1-N3	92.20(7)	F9-P2-F8	91.1(2)	N2-C20-C21	121.8(2)	C38-C37-C36	118.3(2)
N1-Ir1-N4	93.59(8)	F9-P2-F10	91.44(11)	C21-C20-C19	121.9(2)	N5-C37-C36	117.6(3)
N3-Ir1-N4	76.14(8)	F9-P2-P7	179.2(2)	C1-C2-C3	121.3(2)	N5-C37-C38	123.8(3)
C14-Ir1-N2	81.44(9)	F9-P2-P6	92.3(2)	N3-C31-C32	116.1(2)	C25-C26-C21	121.3(2)
C14-Ir1-N1	90.50(8)	C24-N2-Ir1	127.48(17)	N3-C31-C30	121.0(3)	C20-C21-C26	117.0(3)
C14-Ir1-N3	177.26(9)	C24-N2-C20	118.9(2)	C30-C31-C32	122.9(3)	C22-C21-C20	117.7(2)
C14-Ir1-N4	104.14(9)	C20-N2-Ir1	113.60(16)	C7-C6-C1	116.7(2)	C22-C21-C26	125.3(2)
C1-Ir1-N2	93.83(9)	C7-N1-Ir1	112.96(16)	C5-C6-C1	123.7(2)	C16-C17-C18	119.5(2)
C1-Ir1-N2	81.32(9)	C11-N1-Ir1	128.37(18)	C5-C6-C7	119.6(2)	C9-C10-C11	119.9(3)
C1-Ir1-N3	95.60(9)	C11-N1-C7	118.4(2)	N1-C11-C10	122.3(3)	C12-C13-C8	121.3(3)
C1-Ir1-N4	170.21(8)	C31-N3-Ir1	116.21(18)	N3-C27-C28	121.7(3)	C34-C33-C32	120.2(3)
C1-Ir1-C14	84.36(9)	C27-N3-Ir1	124.59(18)	C7-C8-C13	117.1(3)	C26-C25-C18	122.3(2)
F5-P1-F5'	179.33(16)	C27-N3-C31	119.2(2)	C9-C8-C7	117.4(3)	C29-C28-C27	119.3(3)
F2-P1-F5	89.74(7)	C36-N4-Ir1	128.12(17)	C9-C8-C13	125.6(3)	C37-N5-C41	116.2(3)
F3-P1-F5	90.19(6)	C36-N4-C32	117.8(2)	N4-C36-C37	119.4(2)	C10-C9-C8	119.8(2)
F3-P1-F2	87.63(17)	C32-N4-Ir1	112.44(18)	N4-C36-C35	122.0(3)	C29-C30-C31	119.6(3)
F3-P1-F4	88.3(2)	C19-C14-Ir1	112.16(17)	C35-C36-C37	118.3(3)	C4-C3-C2	121.1(3)
F4-P1-F5	90.28(7)	C15-C14-Ir1	131.72(18)	C37-C38-C39	117.8(3)	C17-C16-C15	122.2(3)
F4-P1-F2	175.9(2)	C15-C14-C19	116.0(2)	N4-C32-C31	116.0(2)	C34-C35-C36	119.5(3)
F1-P1-F5	89.81(6)	C20-C19-C14	116.4(2)	N4-C32-C33	121.1(3)	C23-C22-C21	119.9(2)
F1-P1-F2	93.0(2)	C18-C19-C14	123.7(2)	C33-C32-C31	122.9(3)	C3-C4-C5	120.3(3)
F1-P1-F3	179.4(2)	C18-C19-C20	119.8(2)	C14-C15-C16	120.8(3)	C40-C39-C38	119.5(3)
F1-P1-F4	91.1(3)	C2-C1-Ir1	130.99(18)	C13-C12-C5	121.9(3)	C28-C29-C30	119.3(3)
F8-P2-F10	89.89(9)	C2-C1-C6	116.3(2)	C19-C18-C25	117.6(3)	C35-C34-C33	118.8(3)
F8-P2-F7	88.06(19)	C6-C1-Ir1	112.67(18)	C17-C18-C19	117.7(2)	C41-C40-C39	118.9(3)
F10-P2-F10'	177.1(2)	N1-C7-C6	115.9(2)	C17-C18-C25	124.7(2)	C40-C41-N5	123.5(4)
F10-P2-F7	88.56(11)	N1-C7-C8	122.2(2)	C22-C23-C24	119.6(3)		

Table S11. Structural parameters of crystal structure of **3** (bond lengths: Å, bond angles: °).

bond length (Å)							
Ir1-N2	2.037(3)	C7-C6	1.479(6)	C14-C13	1.408(6)	C9-C10	1.371(5)
Ir1-N1	2.044(3)	N5-C41	1.352(6)	C6-C5	1.401(6)	C19-C18	1.381(6)
Ir1-N3	2.126(3)	N5-C45	1.332(6)	C2-C3	1.384(6)	C33-C32	1.371(7)
Ir1-N4	2.246(4)	O1-C46	1.430(6)	C29-C28	1.412(6)	C33-C34	1.380(7)
Ir1-C1	1.999(5)	C15-C14	1.354(6)	C31-C32	1.379(6)	C25-C26	1.394(7)
Ir1-C16	2.008(4)	C22-C21	1.473(6)	C17-C18	1.384(6)	C10-C11	1.400(7)
N2-C22	1.356(5)	C22-C23	1.435(5)	C5-C4	1.372(6)	C38-C39	1.368(6)
N2-C30	1.365(5)	C8-C13	1.433(6)	C13-C12	1.422(6)	C27-C26	1.361(6)
N1-C7	1.355(6)	C8-C9	1.415(6)	C24-C23	1.413(6)	C3-C4	1.393(6)
N1-C15	1.365(6)	C21-C16	1.417(6)	C24-C25	1.364(6)	C12-C11	1.357(7)
N3-C35	1.360(6)	C21-C20	1.400(6)	C20-C19	1.382(6)	C44-C45	1.371(8)
N3-C31	1.344(6)	C36-C35	1.469(6)	C42-C41	1.394(6)	C44-C43	1.388(8)
N4-C36	1.371(5)	C36-C37	1.383(6)	C42-C43	1.378(7)	P1-F5	1.600(3)
N4-C40	1.358(6)	C35-C34	1.389(6)	C28-C23	1.431(6)	P1-F4	1.610(3)
C1-C6	1.410(6)	C16-C17	1.406(6)	C28-C27	1.410(6)	P1-F3	1.608(3)
C1-C2	1.400(6)	C30-C29	1.355(6)	C41-C40	1.470(7)	P1-F6	1.602(3)
C7-C8	1.436(5)	C37-C38	1.374(7)	C40-C39	1.398(6)	P1-F1	1.599(3)
bond angles (°)							
N2-Ir1-N1	175.61(13)	C45-N5-C41	116.8(4)	C12-C13-C8	119.8(4)	N5-C45-C44	124.2(5)
N2-Ir1-N3	99.21(13)	C14-C15-N1	122.1(4)	C25-C24-C23	121.6(4)	C42-C43-C44	118.8(5)
N2-Ir1-N4	83.77(13)	N2-C22-C21	112.5(3)	C19-C20-C21	121.1(4)	F5-P1-F4	89.73(16)
N1-Ir1-N3	83.33(13)	N2-C22-C23	119.6(4)	C43-C42-C41	118.6(5)	F5-P1-F3	179.23(15)
N1-Ir1-N4	100.37(13)	C23-C22-C21	127.9(4)	C29-C28-C23	117.7(4)	F5-P1-F6	90.31(15)
N3-Ir1-N4	75.71(14)	C13-C8-C7	117.7(4)	C27-C28-C29	122.3(4)	F3-P1-F4	89.76(15)
C1-Ir1-N2	96.29(15)	C9-C8-C7	125.7(4)	C27-C28-C23	120.0(4)	F6-P1-F4	89.67(15)
C1-Ir1-N1	79.81(16)	C9-C8-C13	116.6(4)	N5-C41-C42	122.9(4)	F6-P1-F3	89.12(15)
C1-Ir1-N3	96.70(16)	C16-C21-C22	114.0(4)	N5-C41-C40	115.3(4)	F1-P1-F5	90.46(15)
C1Ir1-N4	172.29(14)	C20-C21-C22	125.7(4)	C42-C41-C40	121.6(4)	F1-P1-F4	89.79(16)
C1-Ir1-C16	83.03(16)	C20-C21-C16	120.0(4)	N4-C40-C41	120.7(4)	F1-P1-F3	90.12(14)
C16-Ir1-N2	79.31(14)	N4-C36-C35	115.9(4)	N4-C40-C39	122.1(4)	F1-P1-F6	179.07(16)
C16-Ir1-N1	98.11(14)	N4-C36-C37	122.5(4)	C39-C40-C41	117.2(4)	F2-P1-F5	90.38(16)
C16-Ir1-N3	178.46(14)	C37-C36-C35	121.6(4)	C10-C9-C8	121.9(4)	F2-P1-F4	179.77(18)
C16-Ir1-N4	104.51(15)	N3-C35-C36	116.2(4)	C18-C19-C20	118.9(4)	F2-P1-F3	90.12(15)
C22-N2-Ir1	117.1(3)	N3-C35-C34	120.5(4)	C32-C33-C34	119.7(4)	F2-P1-F6	90.13(16)
C22-N2-C30	120.5(4)	C34-C35-C36	123.3(4)	C24-C23-C22	124.6(4)	F2-P1-F1	90.41(15)
C30-N2-Ir1	122.4(3)	C21-C16-Ir1	115.4(3)	C24-C23-C28	116.9(4)		
C7-N1-Ir1	116.9(3)	C17-C16-Ir1	126.4(3)	C28-C23-C22	118.5(4)		
C7-N1-C15	120.5(4)	C17-C16-C21	117.8(4)	C19-C18-C17	121.6(4)		
C15-N1-Ir1	122.3(3)	C29-C30-N2	121.9(4)	C24-C25-C26	120.6(4)		
C35-N3-Ir1	117.6(3)	C38-C37-C36	120.0(4)	C33-C32-C31	118.7(5)		
C31-N3-Ir1	122.9(3)	C15-C14-C13	120.3(4)	C9-C10-C11	120.3(5)		
C31-N3-C35	119.0(4)	C1-C6-C7	114.4(4)	C39-C38-C37	118.5(4)		
C36-N4-Ir1	112.6(3)	C5-C6-C1	119.4(4)	C26-C27-C28	120.5(4)		
C40-N4-Ir1	128.9(3)	C5-C6-C7	126.2(4)	C33-C34-C35	119.6(5)		
C40-N4-C36	116.7(4)	C3-C2-C1	121.4(4)	C2-C3-C4	119.9(4)		

C6-C1-Ir1	115.4(3)	C30-C29-C28	120.7(4)	C38-C39-C40	120.1(5)	
C2-C1-Ir1	126.4(3)	N3-C31-C32	122.5(5)	C27-C26-C25	120.3(4)	
C2-C1-C6	118.1(4)	C18-C17-C16	120.6(4)	C5-C4-C3	119.5(5)	
N1-C7-C8	120.1(4)	C4-C5-C6	121.3(4)	C11-C12-C13	120.5(5)	
N1-C7-C6	112.7(3)	C14-C13-C8	118.2(4)	C12-C11-C10	120.4(4)	
C8-C7-C6	127.1(4)	C14-C13-C12	121.9(4)	C45-C44-C43	118.7(5)	

Table S12. Structural parameters of crystal structure of **4** (bond lengths: Å, bond angles: °).

bond length (Å)							
Ir1-N2	2.043(3)	C14-C19	1.409(5)	C33-C34	1.377(6)	C42-C43	1.390(7)
Ir1-N4	2.202(3)	C14-C15	1.394(5)	C16-C17	1.389(5)	C42-C48	1.480(7)
Ir1-N3	2.127(3)	C32-C31	1.470(5)	C25-C24	1.381(5)	C42-C47	1.374(7)
Ir1-N1	2.065(3)	C32-C33	1.391(5)	C28-C29	1.383(5)	C43-C44	1.372(9)
Ir1-C1	2.008(3)	C19-C18	1.396(5)	C28-C27	1.375(5)	C50-C49	1.384(7)
Ir1-C14	2.016(3)	C19-C20	1.433(5)	C30-C29	1.379(6)	C50-C51	1.349(8)
S2-C20	1.719(3)	C18-C17	1.379(5)	C36-C35	1.394(5)	C49-C54	1.348(8)
S2-C21	1.739(4)	C26-C21	1.399(5)	C36-C37	1.487(5)	C49-C55	1.494(8)
S1-C7	1.722(4)	C26-C25	1.391(5)	C22-C23	1.377(5)	C51-C52	1.361(9)
S1-C8	1.729(5)	C31-C30	1.376(5)	C12-C11	1.381(5)	C47-C46	1.395(8)
N2-C20	1.323(4)	C15-C16	1.394(5)	C24-C23	1.396(6)	C45-C44	1.388(11)
N2-C26	1.392(4)	N5-C37	1.351(5)	C35-C34	1.364(5)	C45-C46	1.365(11)
N4-C32	1.364(4)	N5-C41	1.350(5)	C37-C38	1.368(5)	C52-C53	1.391(9)
N4-C36	1.342(5)	C21-C22	1.384(5)	C38-C39	1.377(6)	C54-C53	1.377(9)
N3-C31	1.352(4)	C6-C7	1.433(5)	C41-C40	1.378(6)	P1-F2	1.588(3)
N3-C27	1.342(5)	C6-C5	1.394(5)	C40-C39	1.366(7)	P1-F3	1.586(3)
N1-C13	1.402(5)	C13-C12	1.379(6)	C4-C5	1.377(7)	P1-F1	1.578(3)
N1-C7	1.311(5)	C13-C8	1.398(5)	C8-C9	1.395(6)	P1-F4	1.575(3)
C1-C6	1.403(5)	C2-C3	1.395(5)	C10-C11	1.386(7)	P1-F6	1.598(3)
C1-C2	1.392(5)	C3-C4	1.378(6)	C10-C9	1.356(7)	P1-F5	1.563(4)
bond angles (°)							
N2-Ir1-N4	92.24(10)	C15-C14-Ir1	129.3(3)	C24-C25-C26	118.1(3)	C47-C42-C43	119.6(5)
N2-Ir1-N3	100.81(11)	C15-C14-C19	115.9(3)	C27-C28-C29	118.9(4)	C47-C42-C48	119.9(5)
N2-Ir1-N1	168.04(10)	N4-C32-C31	117.1(3)	C31-C30-C29	119.8(3)	C10-C9-C8	118.4(4)
N3-Ir1-N4	75.97(11)	N4-C32-C33	121.7(3)	N4-C36-C35	122.0(3)	C44-C43-C42	119.4(6)
N1-Ir1-N4	97.23(11)	C33-C32-C31	121.1(3)	N4-C36-C37	119.9(3)	C51-C50-C49	120.0(5)
N1-Ir1-N3	88.60(11)	C14-C19-C20	112.5(3)	C35-C36-C37	118.2(3)	C50-C49-C55	120.0(6)
C1-Ir1-N2	92.12(12)	C18-C19-C14	122.9(3)	N1-C7-S1	115.3(3)	C54-C49-C50	118.3(5)
C1-Ir1-N4	168.78(12)	C18-C19-C20	124.6(3)	N1-C7-C6	118.8(3)	C54-C49-C55	121.7(6)
C1-Ir1-N3	93.06(12)	C17-C18-C19	119.4(3)	C6-C7-S1	125.9(3)	C50-C51-C52	121.8(5)
C1-Ir1-N1	79.91(13)	N2-C20-S2	115.2(3)	C30-C29-C28	118.7(3)	C42-C47-C46	120.4(6)
C1-Ir1-C14	83.10(13)	N2-C20-C19	118.5(3)	C23-C22-C21	117.6(3)	C46-C45-C44	119.3(5)
C14-Ir1-N2	79.89(12)	C19-C20-S2	126.3(3)	N3-C27-C28	122.6(3)	C51-C52-C53	119.2(5)
C14-Ir1-N4	107.84(11)	N2-S26-C21	113.3(3)	C18-C17-C16	119.2(3)	C49-C54-C53	122.8(5)
C14-Ir1-N3	176.13(11)	C25-C26-N2	127.1(3)	C13-C12-C11	119.0(4)	C54-C53-C52	117.8(5)
C14-Ir1-N1	90.24(12)	C25-C26-C21	119.5(3)	C25-C24-C23	121.7(3)	C43-C44-C45	121.2(6)
C20-S2-C21	89.52(16)	N3-C31-C32	115.0(3)	C34-C35-C36	119.8(4)	C45-C46-C47	120.0(6)
C7-S1-C8	89.38(19)	N3-C31-C30	121.5(3)	N5-C37-C36	115.7(3)	F2-P1-F6	88.68(18)
C20-N2-Ir1	113.6(2)	C30-C31-C32	123.5(3)	N5-C37-C38	125.0(3)	F3-P1-F2	89.3(2)
C20-N2-C26	111.7(3)	C14-C15-C16	121.5(3)	C38-C37-C36	119.2(3)	F3-P1-F6	87.2(2)
C26-N2-Ir1	134.5(2)	C41-N5-C37	115.2(3)	C22-C23-C24	120.7(3)	F1-P1-F2	90.99(17)
C32-N4-Ir1	112.7(2)	C26-C21-S2	110.3(3)	C37-C38-C39	117.9(4)	F1-P1-F3	89.71(19)
C36-N4-Ir1	128.4(2)	C22-C21-S2	127.4(3)	N5-C41-C40	123.3(4)	F1-P1-F6	176.9(2)
C36-N4-C32	118.0(3)	C22-C21-C26	122.3(3)	C39-C40-C41	119.4(4)	F4-P1-F2	179.3(2)
C31-N3-Ir1	117.1(2)	C1-C6-C7	112.9(3)	C5-C4-C3	119.4(4)	F4-P1-F3	91.0(2)
C27-N3-Ir1	124.2(2)	C5-C6-C1	122.5(4)	C4-C5-C6	119.3(4)	F4-P1-F1	89.64(18)
C27-N3-C31	118.4(3)	C5-C6-C7	124.6(4)	C35-C34-C33	119.1(3)	F4-P1-F6	90.71(18)
C13-N1-Ir1	134.3(2)	C12-C13-N1	128.1(3)	C13-C8-S1	110.9(3)	F5-P1-F2	90.0(2)
C7-N1-Ir1	113.3(2)	C12-C13-C8	119.4(4)	C9-C8-S1	128.0(4)	F5-P1-F3	179.2(2)

C7-N1-C13	111.9(3)	C8-C13-N1	112.5(4)	C9-C8-C13	121.1(4)	F5-P1-F1	90.6(2)
C6-C1-Ir1	114.8(3)	C1-C2-C3	120.5(4)	C9-C10-C11	121.1(4)	F5-P1-F4	89.7(3)
C2-C1-Ir1	128.2(3)	C4-C3-C2	121.5(4)	C12-C11-C10	121.0(4)	F5-P1-F6	92.4(3)
C2-C1-C6	116.9(3)	C34-C33-C32	119.2(3)	C40-C39-C38	119.2(4)		
C19-C14-Ir1	114.4(2)	C17-C16-C15	121.0(3)	C43-C42-C48	120.5(5)		
